# DNA damage accumulates and responses are engaged in human ALS brain and spinal motor neurons and DNA repair is activatable in iPSC-derived motor neurons with SOD1 mutations

**DOI:** 10.1186/s40478-019-0874-4

**Published:** 2020-01-31

**Authors:** Byung Woo Kim, Ye Eun Jeong, Margaret Wong, Lee J. Martin

**Affiliations:** 1 Department of Pathology, Johns Hopkins University School of Medicine, 558 Ross Building, 720 Rutland Avenue, Baltimore, MD 21205-2196 USA; 2 Division of Neuropathology, the Pathobiology Graduate Training Program, Johns Hopkins University School of Medicine, Baltimore, MD USA; 3 Department of Neurology, Johns Hopkins University School of Medicine, Baltimore, MD USA; 4 Department of Neuroscience, Johns Hopkins University School of Medicine, Baltimore, MD USA

**Keywords:** Apoptosis, ATM, Motor cortex, Motor neuron, DNA *N*-glycosylase, Base excision repair, p53

## Abstract

DNA damage is implicated in the pathogenesis of amyotrophic lateral sclerosis (ALS). However, relationships between DNA damage accumulation, DNA damage response (DDR), and upper and lower motor neuron vulnerability in human ALS are unclear; furthermore, it is unknown whether epigenetic silencing of DNA repair pathways contributes to ALS pathogenesis. We tested the hypotheses that DNA damage accumulates in ALS motor neurons along with diminished DDR, and that DNA repair genes undergo hypermethylation. Human postmortem CNS tissue was obtained from ALS cases (*N* = 34) and age-matched controls without neurologic disease (*N* = 15). Compared to age-matched controls, abasic sites accumulated in genomic DNA of ALS motor cortex and laser capture microdissection-acquired spinal motor neurons but not in motor neuron mitochondrial DNA. By immunohistochemistry, DNA damage accumulated significantly in upper and lower motor neurons in ALS cases as single-stranded DNA and 8-hydroxy-deoxyguanosine (OHdG) compared to age-matched controls. Significant DDR was engaged in ALS motor neurons as evidenced by accumulation of c-Abl, nuclear BRCA1, and ATM activation. DNA damage and DDR were present in motor neurons at pre-attritional stages and throughout the somatodendritic attritional stages of neurodegeneration. Motor neurons with DNA damage were also positive for activated p53 and cleaved caspase-3. Gene-specific promoter DNA methylation pyrosequencing identified the DNA repair genes *Ogg1*, *Apex1*, *Pnkp* and *Aptx* as hypomethylated in ALS. In human induced-pluripotent stem cell (iPSC)-derived motor neurons with familial ALS SOD1 mutations, DNA repair capacity was similar to isogenic control motor neurons. Our results show that vulnerable neurons in human ALS accumulate DNA damage, and contrary to our hypothesis, strongly activate and mobilize response effectors and DNA repair genes. This DDR in ALS motor neurons involves recruitment of c-Abl and BRCA1 to the nucleus in vivo, and repair of DNA double-strand breaks in human ALS motor neurons with SOD1 mutations in cell culture.

## Introduction

DNA damage contributes to the mechanisms of aging and has broad relevance to many human cancers, aging, premature aging syndromes, and some neurological disorders [52, 66]. Phenomena involving DNA damage are so important that more than 125 genes in the human genome encode products involved directly in DNA repair [104, 136]. DNA damage, abnormalities in DNA repair, and other nuclear abnormalities are implicated in the pathogenesis of human amyotrophic lateral sclerosis (ALS) [7, 47, 48, 61, 65, 66, 102]. ALS is fatal; patients will die from skeletal muscle paralysis, wasting, and respiratory failure typically 3 to 5 years after diagnosis [107, 141], and it is the third most common adult-onset neurodegenerative disease. Aging is a major risk factor of ALS [67, 107, 141], and human brain aging is associated with increased oxidative damage to DNA [59, 85]. 8-hydroxy-deoxyguanosine (OHdG) levels, a signature of oxidative damage to DNA [27], are elevated in postmortem CNS tissue extracts from individuals with ALS [26]. DNA damage in ALS is caused possibly by oxidative stress from mitochondrial or superoxide dismutase-1 dysfunction [3, 6, 78]. DNA damage as an upstream pathogenic event in human ALS is supported by p53 activation and its import into the nucleus of motor neurons [64], widespread brain activation of poly (ADP-ribose) polymerase [48], and hyperactivation and nuclear accumulation of apurinic/apyrimidinic endodeoxyribonuclease-1 [111].

Some causal genetic factors related to DNA maintenance and repair biology have been suggested in human ALS. Dominant missense mutations in the *senataxin* gene, encoding a DNA/RNA helicase, link to juvenile ALS (ALS4) [13, 91]. Missense mutations in the *apurinic/apyrimidinic endodeoxyribonuclease-1* (*Apex1*) gene have been identified in sporadic and familial ALS [97], though other studies have not identified prominent contributions of *Apex1* mutations to ALS [39, 119]. A Ser326Cys polymorphism in 8-oxoguanine DNA glycosylase (*Ogg1*), the enzyme responsible for excision of 8-oxoguanine, is associated with sporadic ALS [16] but not with Alzheimer’s disease [17]. This gene polymorphism is meaningful etiologically to human disease because this isoform of OGG1 has diminished capacity to repair oxidatively damaged DNA [127]. More recently, DNA damage is a possible mechanism of disease in familial ALS linked to C9orf72 repeat expansions in cell culture [23]. In mice, enforced DNA repair can strongly protect against spinal motor neuron degeneration caused by axonal injury [83]. However, the different forms of DNA damage that accumulate in human ALS are not characterized fully and the specific neural cell types vulnerable to DNA damage in ALS are uncertain; moreover, mechanisms of DNA damage accumulation in human ALS neurons are not understood. Possible mechanisms for elevated levels of DNA damage include mutant protein-related interference in DDR, augmented production of genotoxic stressors, faulty DNA damage response (DDR), and epigenetic silencing of DNA repair genes [66, 72, 82].

In this study, we used human postmortem tissue and human induced pluripotent stem cell (iPSC)-derived motor neurons with familial ALS-causing *superoxide dismutase-1* (*SOD1*) mutations to test the hypothesis that ALS motor neurons accumulate genomic DNA lesions and have aberrant DDR and epigenetic silencing of DNA repair enzyme promoters, thus possibly accounting for DNA damage accumulation. We found in ALS diseased motor neurons: 1) DNA damage accumulation; 2) activation of DDR; and 3) demethylation of DNA repair genes, rather than silencing. In human iPSC-derived motor neurons with *SOD1* mutations, DDR and DNA repair appear equivalent to controls. These results show that genomic DNA damage is a potential mechanism for neurodegeneration in ALS and that motor neurons have the capacity to respond to this cytotoxic threat.

## Materials and methods

### Human tissues

CNS tissues (Table 1) were obtained from the Human Brain Resource Center at JHMI. The institutional IRB and Health, Safety & Environment committee (JHU registration B1011021110) approved the use of postmortem human tissues. The protocol met all ethical and safety standards. De-identified postmortem samples of brain (cerebral cortex Brodmann areas 4 and 3) and spinal cord were from patients with either sporadic ALS or familial ALS (Table 1). De-identified aged human control CNS tissues were from individuals without neurological disease (Table 1). Cases of Alzheimer’s disease (AD) were used as neurological disease controls for some immunohistochemical assays to examine whether ALS related changes are disease specific. The group sizes were controls (*n* = 15); ALS cases (*n* = 34); and AD cases (*n* = 10). ALS patients were diagnosed by neurological examination using El Escorial criteria [105, 107]. AD patients were diagnosed as described [30, 114, 124]. The groups were matched for age and postmortem delay (Table 1). Cases were obtained randomly as autopsies occurred, and accessioning was independent of gender and race; therefore, males, females and minorities are represented. Postmortem brain and spinal cord tissues were snap-frozen and stored as unfixed frozen brain slabs and spinal cord segments at -70 °C. The tissues were microdissected, including laser capture microdissection (LCM), for use in biochemical assays for apurinic/apyrimidinic (AP) sites (also called abasic sites), OHdG, promoter specific CpG 5-methylcytosine (5mC), and western blotting for DNA damage response (DDR) proteins. Formalin-fixed, paraffin-processed tissue was used for immunohistochemical studies of DNA damage and DDR protein localization.

**Table 1:** Human Autopsy Cases Used for Brain and Spinal Cord Samples

Group	Case Number	Age (years)/Sex	Postmortem Delay (hours)	Cause of Death
Controls (Neurological Disease Free)	487	73/male	22	Pancreatic cancer
	515	62/male	21	Aortic aneurysm
	712	44/female	20	Pneumonia
	719	66/male	10	Myocardial infarction
	961	59/female	6	Myocardial infarction
	993	66/male	12	Prostatic carcinoma
	1277	80/female	8	Lymphoma
	1344	53/male	12	Metastatic carcinoma
	1348	44/male	18	Lymphoma
	1361	49/female	15	Thromboembolic disease
	1517	71/female	16	Heart disease
	1591	94/male	16	Pneumonia
	1603	89/male	16	Pulmonary embolism
	1613	74/male	4	Myocardial infarction
	1683	91/female	8	Cardiomyopathy
ALS	345	59/female	3	Respiratory arrest
	414	65/male	4	Respiratory arrest
	433	71/male	17	Respiratory arrest
	447	69/female	15	Respiratory arrest
	492	68/female	18	Respiratory arrest
	834F	46/male	3	Respiratory arrest
	875	70/female	24	Respiratory arrest
	950F	38/male	22	Respiratory arrest
	1014	72/male	5	Respiratory arrest
	1088	66/male	7	Respiratory arrest
	1108	64/female	8	Respiratory arrest
	1151	57/female	14	Respiratory arrest
	1161	47/male	6	Pneumonia
	1169	67/female	15	Respiratory arrest
	1176F	27/male	6	Respiratory arrest
	1359	61/female	14	Respiratory arrest
	1365	59/male	7	Respiratory arrest
	1386	69/female	15	Pneumonia
	1413	79/male	10	Respiratory arrest
	1452	65/female	6	Respiratory arrest
	1453	60/male	22	Pneumonia
	1485	61/female	5	Pneumonia
	1589	55/male	10	Pneumonia
	1614	69/male	18	Respiratory arrest
	1620	63/male	10	Respiratory arrest
	1623	71/female	59	Cardiopulmonary arrest
	1629	55/female	12	Pneumonia
	1635	68/female	5	Respiratory arrest
	1668	76/male	7	Respiratory arrest
	1693F	42/male	6	Respiratory arrest
	1713	54/female	14	Respiratory arrest
	1742	55/male	5	Pneumonia
	1755	72/female	5	Respiratory arrest
	1789F-SOD1A4V	55/male	14	Respiratory arrest

### Laser capture microdissection

Blocks of frozen unfixed human spinal cords (lumbosacral and cervical) from ALS and control cases were cut into transverse sections (8 μm) using a cryostat. Sections were collected on glass slides and stored at -70 °C. For LCM the sections were stained faintly with Ponceau S containing protease inhibitors (allows cytoarchitecture to be visualized without damaging macromolecules). This section thickness and staining are optimal for motor neuron visualization and capture onto CapSure LCM caps [32, 78]. Control individuals 50–70 years of age have approximately 55,000 lumbosacral limb motor neurons [120], and, despite the widespread loss of spinal ventral horn motor neurons in ALS, large numbers of motor neurons remain at endstage disease [113] for capture. Motor neurons in ALS cases were captured at the pre-attritional, chromatolytic, and early attritional stages of degeneration [63]. About 8000–10,000 spinal motor neurons were collected from each individual.

### Measurement of AP sites in DNA

Genomic DNA was extracted from motor cortex (Brodmann area 4) and primary somatosensory cortex (Brodmann area 3) gray matter and from LCM-acquired motor neurons using a phenol-chloroform method [63] or a sodium iodide method [35]. For mitochondrial DNA (mtDNA) extraction from motor cortex, subcellular fractions were prepared [137], and DNA was extracted from the mitochondrial pellet and size fractionated by agarose gel electrophoresis (Additional file 1: Figure S1A). Because AP sites are major DNA lesions caused by free radicals [40], DNA-AP sites were measured using a highly sensitive (Additional file 1: Figure S1B) aldehyde reactive probe-based assay (Kamiya Biomedical Company).

### Measurement of OHdG in DNA

Genomic DNA was extracted from motor cortex and primary somatosensory cortex gray matter and ventral horn spinal cord using a sodium iodide method [35]. OHdG was measured using an enzyme-linked immunosorbent assay (Cell Biolabs).

### Gene promoter-specific methylated DNA pyrosequencing

Genomic DNA was extracted from human ALS and control motor cortex and LCM-acquired spinal motor neurons and dorsal horn gray matter. CpG rich regions of promoter sequences were identified (Table 2) in human *Ogg1*, *apurinic/apyrimidinic endodeoxyribonuclease-1* (*Apex1*), *aprataxin* (*Aptx*), and *polynucleotide kinase 3’-phosphatase* (*Pnkp*) using CpG Island Explorer [131]. Purified DNA (2 μg) was bisulfite treated using an Epitek Bisulfite kit (Qiagen). Purified converted DNA (10 ng) was then PCR amplified (primers and conditions designed and supplied by Qiagen using the Pyromark software). Gene target sequences are shown in Table 2. DNA was sequenced using a Pyromark Q24 system [62]. All samples were run in duplicate. As a positive control, human DNA was methylated in vitro with CpG methytransferase (M.SssI) and then pyrosequenced. 5mC content at all CpG sites was nearly 100%. The data was validated by internal controls and presented as percent 5mC/cytosine ± standard deviation with high agreement in duplicate measures.

**Table 2:** Human ALS & Control DNA Methylation Pyrosequencing Targets

**Aprataxin**	
Gene symbol	*APTX*
Entrez Gene ID	54,840
Sequenced strand	Sense
Amplicon size	253
Biotin modification	reverse PCR primer
Sequence to analyze	ACGCAAAGTGGGTCGAAGACCAACGCGAGCGCCCG
Sequence after bisulfite	AYGTAAAGTGGGTYGAAGATTAAYGYGAGYGTTYG
Number of CpG sites	6
Nucleotide dispensation order	TATCGTCAATGATGTCGAGAGTATCGTCGAGTCG
**8-oxoguanine DNA glycosylase**	
Gene symbol	*OGG1*
Entrez Gene ID	4968
Sequenced strand	Sense
Amplicon size	253
Biotin modification	reverse PCR primer
Sequence to analyze	CCGTGTGGGCGAGGCCTTAAGGGTCGTGGTCCTTGTCTGGGCGGGGT
Sequence after bisulfite	TYGTGTGGGYGAGGTTTTAAGGGTYGTGGTTTTTGTTTGGGYGGGGT
Number of CpG sites	4
Nucleotide dispensation order	ATCGTGTAGTCGATGTTAGTCGATGTTGTCTGTCG
**Apurinic/apyrimidinic endonuclease 1**	
Gene symbol	*APEX1*
Entrez Gene ID	328
Sequenced strand	Sense
Amplicon size	92
Biotin modification	reverse PCR primer
Sequence to analyze	GTCCGCGCTGGGCCGCAGCTTTCCGGAGCGT
Sequence after bisulfite	GTTYGYGTTGGGTYGTAGTTTTTYGGAGYGT
Number of CpG sites	5
Nucleotide dispensation order	TGTCGTCGTCTAGTCGATAGTTCGAGTCG
**Polynucleotide kinase 3’-phosphatase**	
Gene symbol	*PNKP*
Entrez Gene ID	11,284
Sequenced strand	Antisense
Amplicon size	98
Biotin modification	reverse PCR primer
Sequence to analyze	CGGAGGATCCAGTCCCCGCTACCGGCCTGAGCCTCGCGT
Sequence after bisulfite	YGGAGGATTTAGTTTTYGTTATYGGTTTGAGTTTYGYGT
Number of CpG sites	5
Nucleotide dispensation order	GTCGAGAGTAGATTCGTCTGATCGTGAGTTCGTCG

### Profiling of DNA damage, DDR, and cell death markers by immunohistochemistry and western blotting

Human ALS and control cases were examined for specific DNA lesions and DDR markers in motor cortex, sensory cortex, and spinal cord neurons using immunohistochemistry and immunoblotting as described [63, 64, 111]. To detect DNA lesions in tissue sections, we used commercially available mouse monoclonal antibodies to OHdG (clone N45.1, Oxis International) generated by Toyokuni et al. [123] and to single-stranded DNA (ssDNA, clone F7-26, Alexis Biochemicals) generated by Frankfurt [28]. These antibodies have been carefully validated by the originators and by us [1, 81]. To detect DDR in CNS tissue sections, we used commercial rabbit polyclonal antibody to phosphorylated c-Abl^Tyr245^ (Cell Signaling Technology) and mouse monoclonal antibody to BRCA1 (clone MS110, Millipore). Immunohistochemical negative controls included concentration-identical substitution of specific primary antibody with non-immune isotype IgG and incubation of sections with no primary antibody but with all other immunoperoxidase-diaminobenzidene (DAB) steps unchanged. Western blotting also validated the DDR antibodies. The BRCA1 antibody specificity was further validated by human BRCA1 viral siRNA knockdown (ABM, Inc) in cultures of a human cortical neuron cell line (HCN1, American Type Culture Collection). Total cell lysates were prepared for western blotting. To identify relationships between DNA lesions and cell death markers we used antibodies to mouse monoclonal antibody to OHdG and rabbit polyclonal antibody to phospho-p53^Ser15^ (Cell Signaling Technology) and OHdG antibody paired with rabbit polyclonal antibody to cleaved caspase-3 (Cell Signaling Technology). These antibodies have been validated [79, 81]. Dual antigen visualization was done with DAB and benzidine dihydrochloride (BDHC) as chromogens [30, 56, 70] to avoid the confounding influence of endogenous lipofuscin and paraffin processing generated autofluorescence [45]. Mitochondrial and cleaved caspase-3 relationships were examined with a mouse monoclonal antibody to cytochrome c oxidase subunit I (clone 1D6-E1-A8, Molecular Probes Invitrogen) that has been validated [71].

Immunohistochemical preparations were analyzed quantitatively using cell counting and single-cell densitometry [68, 73, 111]. The evaluation focused on motor cortex and spinal cord anterior horn of control and ALS cases. In carefully selected sections that were at anatomically matched regions of motor cortex and spinal cord, ssDNA and c-Abl immunoreactive neuronal cell bodies were counted in layer 5 of motor cortex or ventral horn of spinal cord in 15–20 non-overlapping microscopic fields at 400x magnification in at least 3 paraffin sections per case. The sections were counter-stained with cresyl violet to assist in cortical layer, Rexed layer, and cell identifications. Only cells with a discernible nucleus were counted. For OHdG immunoreactivity quantification in control, ALS, and AD tissue sections, grayscale images of randomly selected immunoreactive layer 5 pyramidal neurons in motor cortex and somatosensory cortex and motor neurons in spinal cord were acquired at 1000x magnification by an observer unaware of case history. AD CNS tissue sections were used as a different neurodegenerative disease setting to deterime if identified changes in ALS neurons are disease specific. The sections were not counter-stained until after image acquisition. For each case, approximately 50 neurons were acquired. In ImageJ, each neuronal perikaryal profile was delineated as the region of interest, and measurements of optical density were obtained similar as described [10].

For immunoblotting, homogenates from motor cortex of control and ALS cases and HCN cell lysates were prepared, subjected to SDS-PAGE, transferred to nitrocellulose membranes, and stained with Ponceau S to confirm uniform protein transfer among lanes and for quantitative normalization as described [63, 64, 111]. Snap frozen fresh samples of AD motor cortex were unavailable for western blotting. For human tissue western blots, soluble protein fractions were used. Membranes were immunoblotted for phosphorylated c-Abl^Thr735^ (rabbit polyclonal, Cell Signaling Technology), total c-Abl (mouse monoclonal, clone 24–11, Santa Cruz Biotechnology), phosphorylated^Ser/Thr^-ATM/ATR protein targets (rabbit polyclonal, Cell Signaling Technology), BRCA1 (mouse monoclonal, clone MS110, Millipore) and Ogg1 (rabbit polyclonal, Novus Biologicals). For OGG1 blots, recombinant human OGG1 (Trevigen) was used as a positive control. Antibodies to synaptophysin (mouse monoclonal, clone SY38) and actin (mouse monoclonal, clone C4, Chemicon) were used as loading controls. The secondary antibodies used were goat-anti-rabbit IgG-HRP and goat-anti-mouse IgG-HRP (BioRad) in milk blocker for 2 h at room temperature. Immunoreactive proteins were visualized by enhanced chemiluminescence and exposure of membrane to x-ray film. Films were digitally scanned for densitometry, with target proteins being normalized to ponceau total protein or to actin or synaptophysin immunoreactivities, and figure generation.

### Cell culture

The institutional biosafety committee (JHU registration B1011021110) approved the use of human cells. The protocols met all ethical and safety standards for work on human cells. The human iPSC lines used in this study are identified in Table 3 and were characterized previously [51, 133]. They were maintained on Matrigel-coated plates in StemFlex Medium (Gibco) and passaged every 4–6 days using EDTA or Accutase (Thermo Fisher Scientific). Mouse embryonic fibroblasts (MEFs) were derived from CF-1 mouse embryos at approximately 13.5 days gestation. MEFs were cultured in Dulbecco’s modified Eagle’s medium (DMEM, Corning) supplemented with 10% fetal bovine serum (FBS, Hyclone), 1% Minimum Essential Medium Non-Essential Amino Acids (MEM-NEAA, Gibco), and 1% GlutaMAX (Gibco). Mouse cortical astrocytes were isolated from 3 to 4 postnatal day old CD1 mouse pups as described [109] and cultured in DMEM supplemented with 10% FBS.

**Table 3:** Human Induced Pluripotent Stem Cell Lines Used

iPSC lines (Clone)^a^	Gene	Mutation	Age of Donor	Gender
C3-1	Control	N/A	40	F
C3-1	SOD1	G93A	40	F
GO013	SOD1	A4V	63	F

^a^C3-1 and GO013 were provided by the Song lab (University of Pennsylvania) and the Rothstein lab (JHMI), respectively

### Genome editing of human iPSCs by CRISPR-Cas9 system

Introduction of SOD1-G93A missense mutation by CRISPR-Cas9 genome editing technology was carried out using a healthy control iPSC line (C3-1). Prior to genome editing, alkaline phosphatase live staining (Invitrogen) was performed to verify pluripotency of iPSCs. Cells cultured on Matrigel (Corning) in StemFlex medium were pre-treated with Y-27632 ROCK inhibitor (Cellagen Technology) for 4–5 h and dissociated with Accutase. Cells were resuspended with Cas9 nuclease (Invitrogen), guide RNA (Table 4), and single-stranded DNA donor (Table 4) and electroporated using Neon Transfection System (Invitrogen). After electroporation, cells were plated on Matrigel-coated plates and cultured for 48 h. Cleavage efficiency was determined in a portion of the cells using GeneArt Genomic Cleavage Detection kit (Invitrogen). Remaining cells were passaged and cultured for 48–72 h before performing clonal isolation. Single cells were isolated using Accutase and cultured for approximately 10–12 days. Each clonal cell line was collected and expanded. The genome editing of each clone was confirmed by DNA Sanger sequencing. Genetic off-target effects were also analyzed.

**Table 4:** List of Oligonucleotide Sequences

ID	Sequence (5’-3’)	Purpose
SOD1-G93A-gRNA-fwd	TAATACGACTCACTATAGGAATCTTCAATAGACACA	gRNA DNA template for gRNA synthesis
SOD1-G93A-gRNA-rev	TTCTAGCTCTAAAACATGTGTCTATTGAAGATTC	
SOD1-G93A-ssODN	p-ZOCATCTGATGCTTTTTCATTATTAGGCATGTTGGAGAC TTGGGCAATGTGACTGCTGACAAAGATGCTGTGGCAGAT GTGTCTATTGAAGATTCZET	knock-in donor DNA
OT1-fwd	AGACACTGGCATTTCAGACGTAGG	Off-target analysis
OT1-rev	AGAAGACCCATGATTCCTGGGC	
OT2-fwd	CAAAGGTAGATCGCCAGAGACAAC	
OT2-rev	TGCCTGGTGGGAAGGTCCAT	
OT3-fwd	CAACCGGAGAAGTGACAAGGC	
OT3-rev	CTCAGATCAGTCTCTAGCAATCCC	
OT4-fwd	TCTGTAGTCAGCCTCTGTCCAC	
OT4-rev	CTTTCTGGGTCCCCAGTTTCTAC	
OT5-fwd	GGGCCTATGGAAGTGAACTCAAC	
OT5-rev	ATGTTGCATGTTTCTATGATGGGCCTATCC	
OT6-fwd	TCCTGGATGGTCCTGGACATCA	
OT6-rev	AGCAATGTGTGTCACTCCAGGATC	
OT7-fwd	GCAAATAAGCAACAGACCGAGGAG	
OT7-rev	GCCTCAGCTTTCTCATGTACCATC	

* p: 5’-phosphate, O: Phosphorothioate-C, E: Phosphorothioate-G, Z: Phosphorothioate-T

### Genetic off-target analysis

Potential off-target sites were analyzed by direct DNA sequencing. The top seven candidates were selected based on COSMID web tool [19]. Genomic DNA was isolated from iPSCs using DNeasy Blood & Tissue Kit (Qiagen). PCR amplification around the seven sites was performed and PCR products were sequenced. Primers used are listed in Table 5.

**Table 5:** Summary of Off-Target Analysis^a^

ID	Sequence	Mismatch	Score	Chromosome position	Mutation
OT1	**C**A**AAA**TTCAATAGACACATGGG -- hit GAATCTTCAATAGACACATNGG – query	3	0.53	Chr15:25520090-25,520,111	Not detected
OT2	G**T**AT**A**TTC**C**ATAGACACATGGG -- hit GAATCTTCAATAGACACATNGG – query	3	0.86	Chr4:109681139-109,681,160	Not detected
OT3	**TC**ATCTTCAA**A**AGACACATTGG -- hit GAATCTTCAATAGACACATNGG – query	3	1.08	Chr13:46227046-46,227,067	Not detected
OT4	G**T**ATCTT**G**AA**A**AGACACATGGG -- hit GAATCTTCAATAGACACATNGG – query	3	1.3	Chr11:87945053-87,945,074	Not detected
OT5	GA**T**TCT**A**CAAT**G**GACACATTGG -- hit GAATCTTCAATAGACACATNGG – query	3	1.54	Chr12:24874767-24,874,788	Not detected
OT6	GAAT**T**T**C**CAAT**G**GACACATTGG -- hit GAATCTTCAATAGACACATNGG – query	3	1.58	Chr7:134494940-134,494,961	Not detected
OT7	**A**AATCTT**T**AATA**C**ACACATCGG -- hit GAATCTTCAATAGACACATNGG – query	3	1.78	Chr4:107649971-107,649,992	Not detected

^a^Mismatched bases are in bold

### Differentiation of human iPSCs into motor neurons

Generation of iPSC-derived spinal motor neurons was done using published protocols [9, 22, 84] with some modifications. In brief, iPSCs were passaged onto MEF feeder layers in DMEM/F12 culture medium supplemented with 20% KnockOut Serum Replacement (Gibco), 1% MEM-NEAA, 1% GlutaMAX, 10 ng/mL bFGF (PeproTech), 0.1 mM β-mercaptoethanol (Gibco), and 10 μm Y-27632 ROCK inhibitor. On the next day, the medium was changed to modified N2/B27 medium (DMEM/F12:Neurobasal [1:1], 0.5% N2, 0.5% B27, 0.1 mM ascorbic acid, and 1% GlutaMAX) containing 3 μM CHIR-99021 (Tocris), a glycogen synthase kinase-3 inhibitor, along with the combination of 2 μM SB-431532 (Tocris), a transforming growth factor-β receptor inhibitor, and 2 μM DMH-1 (Tocris), a bone morphogenic protein type I receptor/activin receptor-like kinase-2 (ALK2) inhibitor. iPSCs were cultured in this condition for 6–7 days. The cell clusters were detached with 0.1% (w/v) collagenase IV (Gibco) and plated on Matrigel-coated plates in the same medium supplemented with 1 μM CHIR-99021, 2 μM SB-431532, 2 μM DMH-1, 0.1 μM retinoic acid (RA, Sigma), and 0.5 μM purmorphamine (Stemgent), a hedgehog agonist. After maintaining cell clusters for 6–7 days, they were collected by collagenase IV and further differentiated in ultra-low attachment plates (Corning) containing modified N2/B27 medium with 0.5 μM RA and 0.1 μM purmorphamine and grown in suspension for another 6–7 days. Cell clusters were then singlized with Accutase and plated on Matrigel-coated plates or on mouse primary astrocytes for additional 10 days with 0.5 μM RA, 0.1 μM purmorphamine, 0.1 μM Compound E (Millipore), a Notch pathway inhibitor, and three neurotrophic factors (PeproTech): 10 ng/ml brain-derived neurotrophic factor (BDNF); 10 ng/ml ciliary neurotrophic factor (CNTF); and 10 ng/ml insulin-like growth factor 1 (IGF-1). We also utilized alterative cell culture conditions. For neural patterning, 10 μM SB-431532 and 200 nM LDN-193189 (Stemgent), an ALK2/3 receptor inhibitor, was used. For motor neurons specification, a combination of 10 μM SB-431532 and 200 nM LDN-193189 was used as a substitute for 1 μM CHIR-99021, 2 μM SB-431532, and 2 μM DMH-1. Lastly, for motor neuron differentiation, we sometimes used 0.5 μM RA, 0.1 μM purmorphamine, 5 μM DAPT (Stemgent), a γ-secretase inhibitor, with BDNF, CNTF, and IGF-1. In all instances, neuronal cultures were treated with 50 μm 5-Fluoro-2’-deoxyuridine (Sigma) on the following day of plating for 24 h to inhibit proliferation of any undifferentiated progenitor cells or astrocytes. All culture media in each stage were changed every 2 days. Cultured neurons were immunophenotyped using neuron- and motor neuron-specific antibodies.

### Etoposide treatment of human iPSC-derived motor neurons

Etoposide (Sigma) is a topoisomerase-II inhibitor that was used to cause DNA damage in the form of strand breaks [58, 79]. It was dissolved at 10 mM in DMSO and further diluted to 10 μM in modified N2/B27 medium with 0.5 μM RA, 0.1 μM purmorphamine, 0.1 μM Compound E, 10 ng/ml BDNF, 10 ng/ml CNTF, and 10 ng/ml IGF-1. Medium containing etoposide was added to iPSC-derived motor neurons differentiated on glass coverslips and incubated for 1 h at 37 °C for DNA double-strand formation [58, 79]. For recovery, cells were first washed once with DMEM/F12. Those with 0-h recovery were then fixed, whereas other cells were incubated in fresh differentiation medium without etoposide for 1.5, 4, or 24 h before fixation.

### Immunofluorescence staining

Cells on glass coverslips were fixed in 4% paraformaldehyde for 10 min at room temperature and washed three times with PBS. Fixed cells were first permeabilized with 0.2% Triton X-100 in PBS for 10 min and were subsequently blocked in PBS with 10% donkey serum for 1 h. Following blocking, cells were incubated overnight at 4 °C with primary antibodies diluted in blocking solution. The following primary antibodies were used: chicken polyclonal anti-microtubule–associated protein-2 (MAP 2) (1:5000, Novus Biologicals), mouse monoclonal anti-Islet-1 (1:100, clone 40.2D6, Developmental Studies Hybridoma Bank [DSHB]), mouse monoclonal anti-Hb9 (1:50, clone 81.5C10, DSHB), goat polyclonal anti-choline acetyltransferase (ChAT) (1:100, Millipore), mouse monoclonal anti-TUJ1 (1:2000, clone 5G8, Promega), and rabbit polyclonal anti-γH2A.X (1:400, Cell Signaling). Non-immune IgG isotypes were used as negative controls at concentrations identical to the primary antibodies. After antibody incubations, cells were rinsed in PBS, incubated with secondary antibodies (Alexa-Fluor-488, Alexa-Fluor-594, and Alexa-Fluor-647, ThermoFisher) diluted at 1:500, rinsed in PBS, and then stained with Hoechst 33258 DNA dye for nuclear visualization.

### Data analysis

The sample populations were selected randomly and were normally distributed (i.e., assumptions for parametric analyses were not violated). The analysis of the measurements was done by comparing age-matched control (disease free and AD) values to ALS values with one-way analysis of variance. Subsequent statistical evaluation of significance was done using a two sample Student’s *t*-test.

## Results

### Genomic DNA AP sites are increased in ALS

**Fig. 1:**
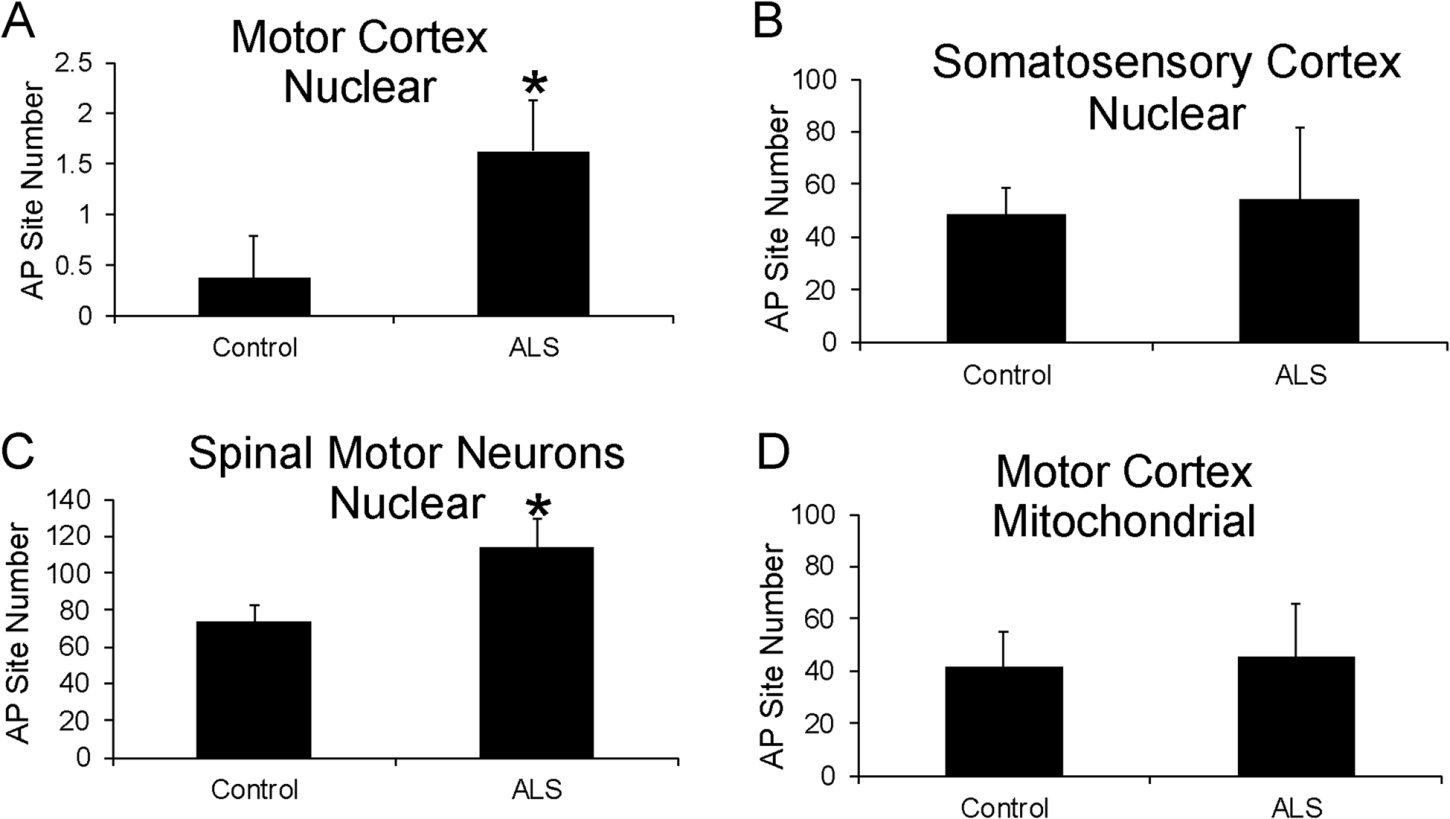
DNA Damage AP Sites Accumulate in Motor Cortex and in Spinal Motor Neurons in Human ALS. **a** AP site number in genomic DNA (100 femtogram) extracted from human ALS (*n* = 16) and age-matched control (*n* = 10) motor cortical gray matter (Brodmann area 4). *p < 0.01. **b** AP site number in genomic DNA (1 picogram) extracted from human ALS (*n* = 16) and age-matched control (*n* = 10) primary somatosensory cortical gray matter (Brodmann area 3). **c** AP site number in genomic DNA (1 picogram) extracted from human ALS (*n* = 16) and age-matched control (*n* = 10) LCM-acquired spinal cord motor neurons (approximately 10,000-16,000 individual neurons). *p < 0.01. **d** AP site number in mitochondrial DNA (100 femtogram) extracted from human ALS (*n* = 16) and age-matched control (*n* = 10) motor cortical gray matter. Values are mean ± SD

AP sites in DNA are very common lesions formed either spontaneously by oxidative stress or as intermediates during DNA repair [2, 52]. They can trigger cell death [49, 128]. We used a highly sensitive biochemical assay to measure AP sites (Additional file 1: Figure S1B) in nuclear DNA and mitochondrial DNA extracted from postmortem human motor cortex, primary somatosensory cortex, and LCM-acquired spinal motor neurons (Fig. 1). AP site number in chromosomal DNA was increased significantly in the motor cortex of ALS cases compared to age-matched controls (Fig. 1a). The number of AP sites in anatomically adjacent somatosensory cortex did not differ among ALS and control (Fig. 1b). In spinal cord, AP sites specifically in the spinal motor neuron genome were significantly elevated in ALS compared to control (Fig. 1c). In contrast, AP sites did not differ in DNA purified from mitochondria isolated from motor cortex of ALS and control individuals (Fig. 1d, Additional file 1: Figure S1A).

### Single-stranded DNA accumulates in ALS upper and lower motor neurons

**Fig. 2:**
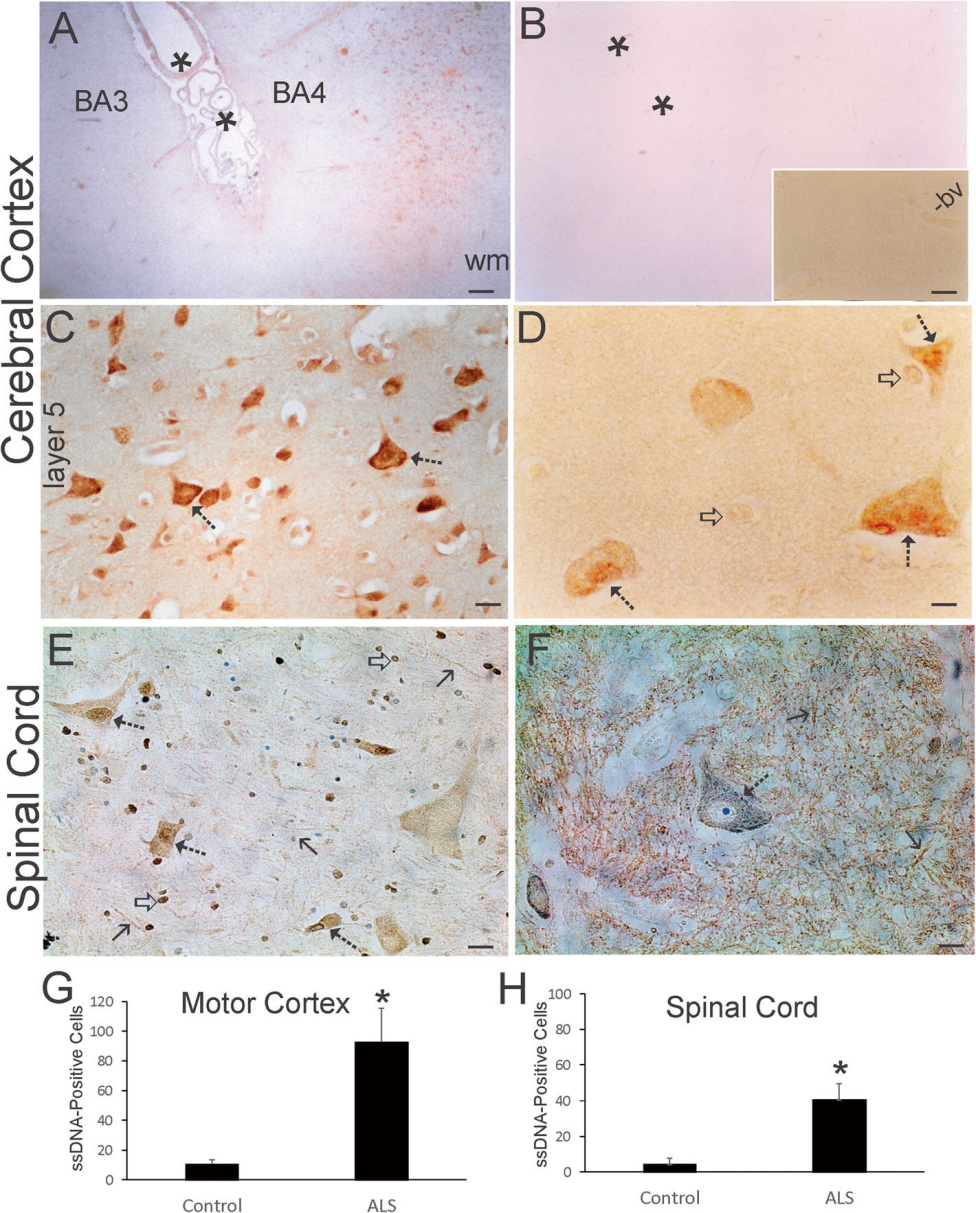
ssDNA Accumulates in Human ALS Upper and Lower Motor Neurons. **a** ssDNA positive profiles (brown) were numerous in ALS motor cortex (Brodmann area 4, BA4) but not in the nearby postcentral gyrus primary somatosensory cortex (Brodmann area 3, BA3). Asterisks identify central sulcus. wm, white matter. **b** Neurologic disease-free age-matched control cerebral cortex had very few pyramidal neurons positive for ssDNA immunoreactivity. Asterisks identify central sulcus. Inset shows higher magnification of control cortical gray matter that is blank. A blood vessel (bv) is a fiducial and shows effective quenching of endogenous peroxidases. **c** In ALS motor cortex, numerous pyramidal neuronal profiles (hatched arrows) were ssDNA-positive (brown), particularly in deep layers. **d** ssDNA immunoreactivity (brown) was localized to the nucleus and cytoplasm of pyramidal neurons (hatched arrows) in ALS motor cortex. Nearby glial cells were either negative or faintly positive (open arrows). **e** Spinal motor neuron cell bodies in ALS cases were positive for ssDNA (hatched arrows); the nucleus was often intensely positive (brown, cresyl violet counterstained). Numerous glial cells were positive (open arrow). Processes in the spinal cord gray matter neuropil were occasionally discernible (solid black arrows). **f** In control spinal cord, most of the ssDNA immunoreactivity (brown, cresyl violet counterstained) was confined to the neuropil, while the motor neuron cell bodies (open arrow) were lightly stained in the cytoplasm and nucleus compared to ALS neurons. **g**. Counts of motor cortical neurons positive for nuclear ssDNA in control (*n* = 8) and ALS (*n* = 14) cases. Values are mean ± SD. *p < 0.001. **h** Counts of ventral horn neurons positive for nuclear ssDNA in control (*n* = 8) and ALS (*n* = 14) cases. Values are mean ± SD. *p < 0.01. Scale bars (in μm) = 140 (A, same for B), 31 (B inset), 14 (C), 5 (D), 30 (E), 44 (F)

AP sites can be converted readily to DNA helix breaks leading to strand gaps and the accumulation of single-stranded DNA [34] and, if closely opposed, to double-strand breaks [37]. We directly visualized ssDNA in human brain using monoclonal antibody F7-26. This antibody has been used widely and characterized extensively [28, 29, 81, 99]. In animal CNS, neurons fated to undergo retrograde degeneration and death, similar to mechanisms proposed in human ALS and rodent models of ALS [63, 76, 78, 83, 138], F7-26 detects the early accumulation of DNA damage in pre-apoptotic neurons [81]. In human ALS, ssDNA strikingly accumulated in motor cortex but not in the anatomically adjacent postcentral gyrus (Fig. 2a, g). Generally non-vulnerable brain regions in ALS cases and aged-matched control cases overall had low or undetectable ssDNA accumulation in cells as visualized by immunohistochemistry (Fig. 2a, b). In contrast, in ALS motor cortex, many pyramidal neurons were ssDNA-positive compared to age-matched controls (Fig. 2c, g). Cortical macroglial cells appeared with very low positivity for ssDNA compared to neurons, where ssDNA accumulated in the perikaryal cytoplasm and nucleus (Fig. 2d). In spinal cord, the pattern of ssDNA staining was different from the telencephalon. In controls, neuropil staining, including processes of neurons and glia, was prominent, but motor neuron cell bodies had low staining (Fig. 2f, h). In ALS spinal cord, the neuropil ssDNA immunoreactivity was markedly attenuated and motor neuron cell body positivity was more abundant (Fig. 2f, h), including strong staining in the nucleus, as we have seen in pre-apoptotic neurons [81].

### c-Abl is upregulated and activated in human ALS CNS

**Fig. 3:**
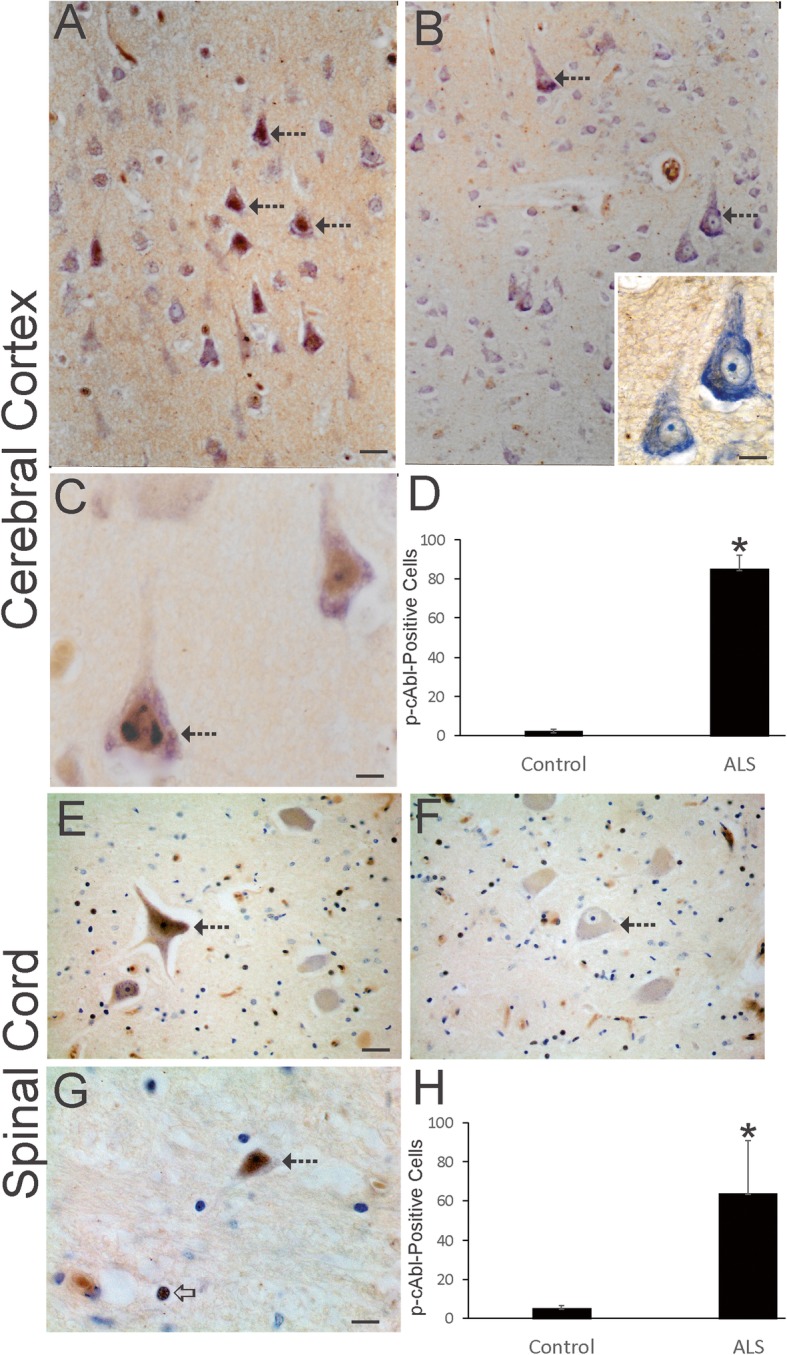
Activated c-Abl Accumulates in Human ALS Upper and Lower Motor Neurons. **a** Many pyramidal neurons (hatched arrows) in ALS motor cortex showed conspicuous accumulation of phosphorylated c-Abl (brown, cresyl violet counterstained). Some pyramidal neurons seen by cresyl violet had low or negative active c-Abl staining. The neuropil also showed immunoreactivity. **b** In control motor cortex, phosphorylated c-Abl (brown, cresyl violet counterstaining) was much less evident in pyramidal neuron cell bodies (hatched arrows) and in the neuropil compared to ALS motor cortex shown in A. Inset shows control motor cortical pyramidal neurons rich in Nissl substance and no active c-Abl immunoreactivity in the nucleus. **c** Phosphorylated c-Abl (brown, cresyl violet counterstaining) was localized to the nucleus of pyramidal neurons (hatched arrows) in ALS motor cortex and was sometimes seen as discrete nuclear inclusions. **d** Counts of motor cortical neurons positive for phosphorylated c-Abl in control (*n* = 8) and ALS (*n* = 16) cases. Values are mean ± SD. *p < 0.001. **e**, **f** Spinal cord motor neurons (hatched arrow) at pre-attritional (not shrunken) stages of degeneration in ALS cases [63] (E, hatched arrow) were strongly positive for phosphorylated c-Abl (brown, cresyl violet counterstain). In control spinal motor neurons (F, hatched arrow) c-Abl immunoreactivty (brown, cresyl violet counterstain) was nearly inconspicuous, even though some surrounding glial cells were positive. **g** Spinal cord motor neurons (hatched arrow) at advanced attritional stages (shrunken) of degeneration in ALS cases [63] (hatched arrow) were strongly positive for phosphorylated c-Abl (brown, cresyl violet counterstain). Subsets of glial cell nucleus were c-Abl-positive (open arrow). **h** Counts of spinal cord ventral horn neurons positive for phosphorylated c-Abl in control (*n* = 8) and ALS (*n* = 16) cases. Values are mean ± SD. *p < 0.001. Scale bars (in μm) = 24 (A, same for B), 10 (B inset), 8 (C), 130 (E, same for F), 8 (G)

Previous studies have reported the activation of p53 and APEX1 and their nuclear accumulation in human ALS motor neurons indicative of a DDR [64, 111] that would be consistent with our AP site (Fig. 1) and ssDNA (Fig. 2) data. To corroborate that DNA damage is accumulating in ALS motor neurons and that appropriate sensor mechanisms are activated, we examined other DDR proteins. c-Abl functions in the human cell DDR where in shuttles from the cytoplasm to the nucleus [60, 115]. Studies in cell culture show that inhibition of c-Abl protects cortical neurons from DNA damage-induced apoptosis [79], revealing that this molecular arm of the DDR can be death-promoting in neurons and consistent with the view that the c-Abl pathway may be a therapeutic target in ALS [44]. In human ALS motor cortex, immunoreactivity for active phosphorylated c-Abl was very robust compared to age-matched control motor cortex (Fig. 3a, b, d). c-Abl immunoreactivity was detected in the neuropil and in neuronal cell bodies (Fig. 3a). Many pyramidal neurons in ALS cerebral cortex were positive compared to controls (Fig. 3d). Active c-Abl was prominent in the nucleus of ALS cortical pyramidal neurons (Fig. 3c) but not in control cortical pyramidal neurons (Fig. 3b inset). c-Abl in ALS cortical pyramidal neuron nuclei was localized diffusely in the nuclear matrix and formed discrete inclusions near the nuclear envelope (Fig. 3c), similar to apoptotic chromatin crescents [65, 67, 69]. In ALS spinal cord, motor neurons were strongly positive for active c-Abl at pre-attritional (Fig. 3e) and attritional (Fig. 3g) stages of degeneration as defined before [40] and were numerous (Fig. 3h), while spinal motor neurons in age-matched controls were scarcely positive for c-Abl (Fig. 3f, h).

**Fig. 4:**
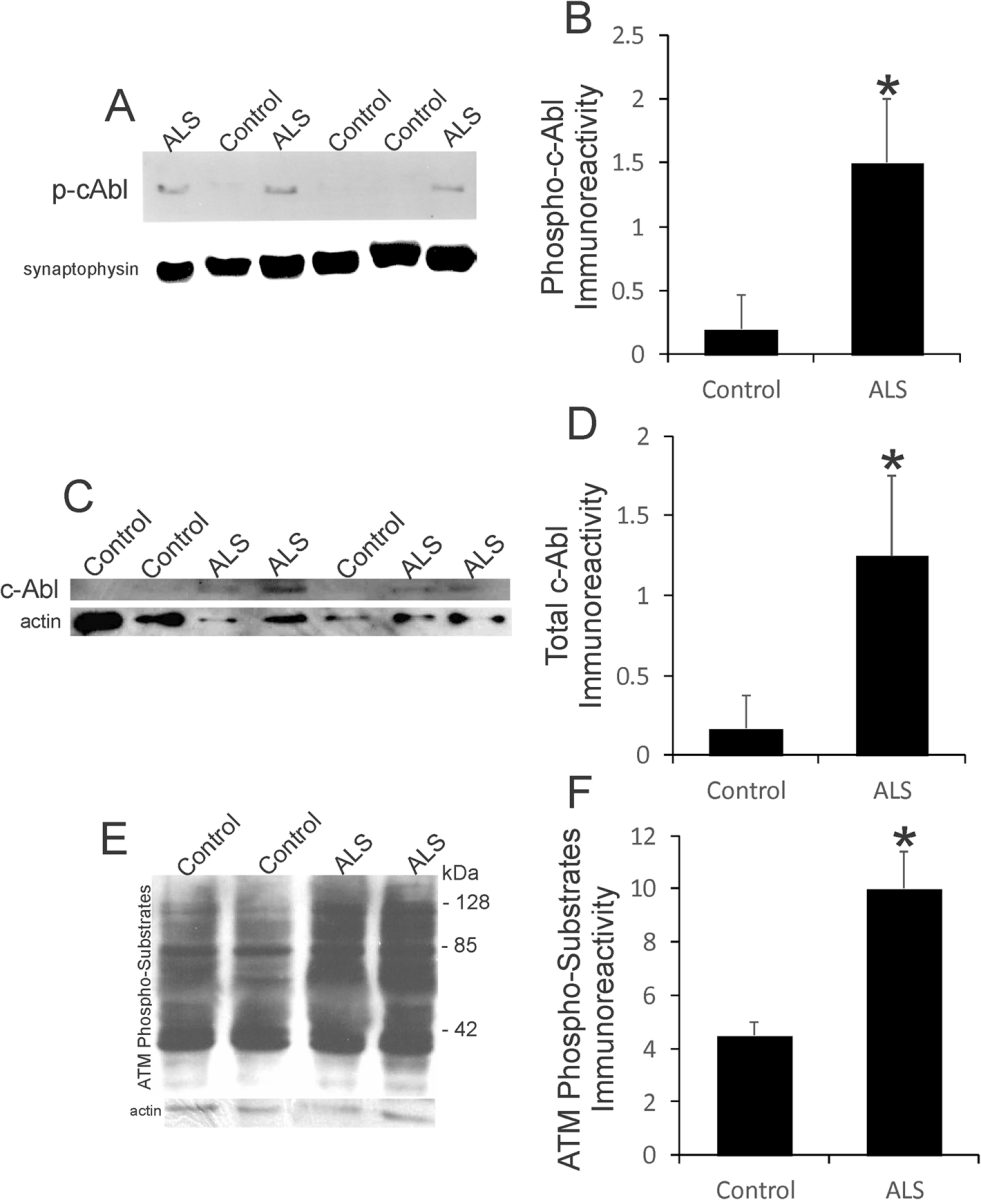
DNA Damage Sensor Kinases are Upregulated and Activated in Human ALS Brain. **a** Western blot for phosphorylated c-Abl in motor cortex homogenates of ALS and age-match controls. Synaptophysin was used as a loading control. **b** Western blot quantification of phosphorylated c-Abl immunoreactivity in (*n* = 8) and ALS (*n* = 12) cases. Values are mean ± SD. *p < 0.001. Western blot for phosphorylated c-Abl in motor cortex homogenates of ALS and age-match controls. Synaptophysin was used as a loading control. **c** Western blot for total c-Abl in motor cortex homogenates of ALS and age-match controls. Actin was used as a loading control. **d** Western blot quantification of total c-Abl immunoreactivity in (*n* = 8) and ALS (*n* = 12) cases. Values are mean ± SD. *p < 0.01. **e** Western blot for phosphorylated targets of ATM in motor cortex homogenates of ALS and age-match controls. Blot probed for actin shows loading. **f** Western blot quantification of ATM phosphorylated target protein immunoreactivity in (*n* = 8) and ALS (*n* = 12) cases. Values are mean ± SD. *p < 0.001

Western blotting corroborated the immunohistochemical findings (Fig. 4). Phospho-c-Abl^Tyr245^ antibody was highly specific at detecting an immunoreactive band at approximately 120 kDa in human brain extracts (Fig. 4a). Active c-Abl was elevated significantly (*p* < 0.001) in ALS motor cortex compared to age-matched controls that showed low levels (Fig. 4a, b). Total c-Abl immunoreactivity was increased significantly (*p* < 0.01) also in ALS motor cortex (Fig. 4c, d), demonstrating that c-Abl was generally upregulated and activated in ALS. Consistent with the activation of c-Abl, an ATM kinase target [129], was the observation that phosphorylated targets of ATM were overall increased in ALS motor cortex compared to age-matched control (Fig. 4e). Immunoreactivity for ATM phosphorylated target proteins in ALS motor cortex was increased significantly (p < 0.001), essentially double, that of control motor cortex (Fig. 4f). Accumulation of ATM/ATR phosphorylated protein targets is an early characteristic of preapoptotic cortical neurons with DNA damage [79].

### BRCA1 is upregulated prominently in human ALS CNS

**Fig. 5:**
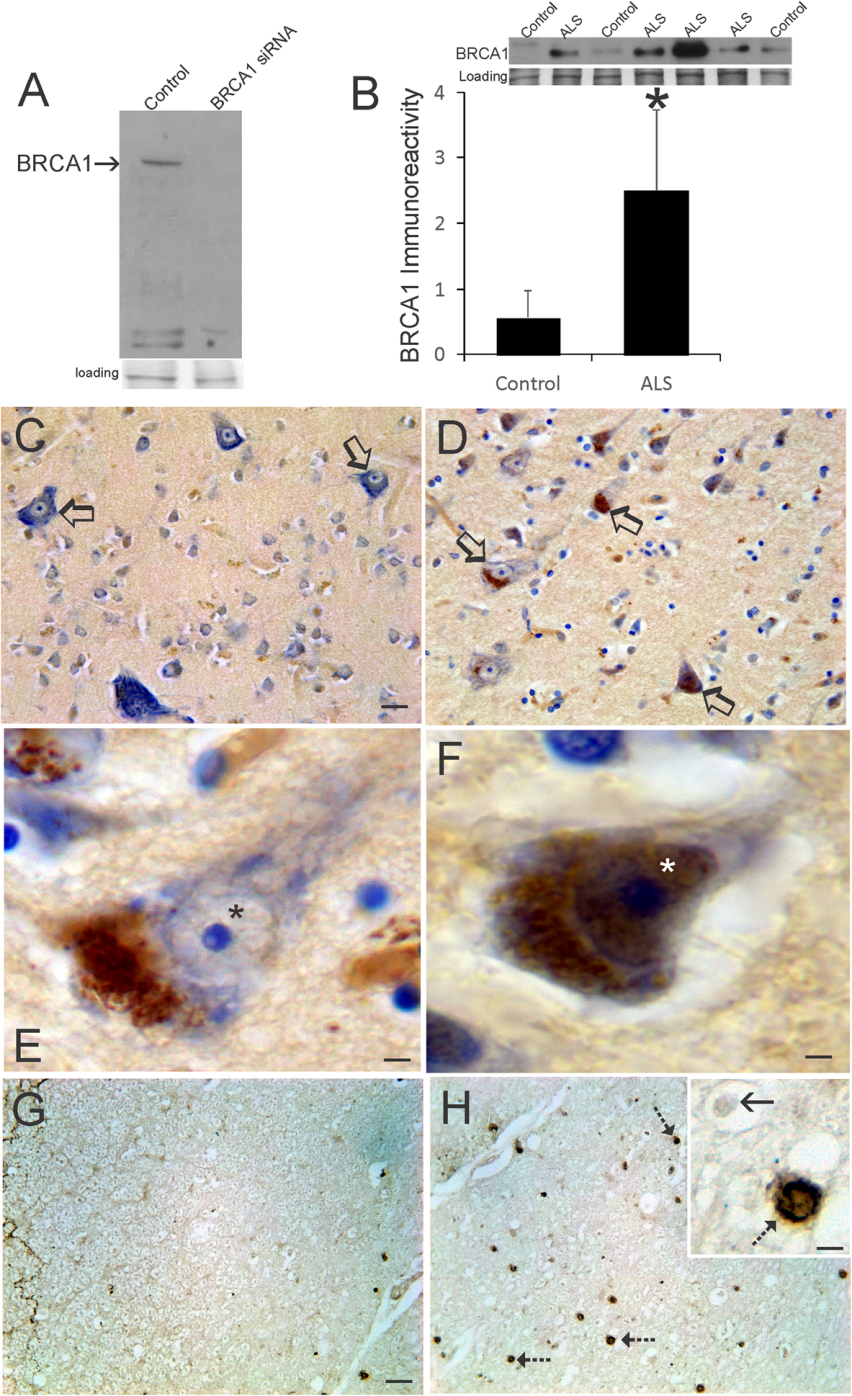
BRCA1 is Upregulated in Human ALS Brain. **a** BRCA1 antibody validation by western blotting after siRNA knockdown in the human cortical neuron cell line HCN1. Ponceau staining of membrane shows protein loading. **b** Western blot for BRCA1 in motor cortex homogenates of ALS and age-match controls. Ponceau staining of membrane shows protein loading. Graph shows quantification of BRCA1 immunoreactivity in (*n* = 8) and ALS (*n* = 12) cases. Values are mean ± SD. *p < 0.01. **c** Immunohistochemical staining for BRCA1 (brown, cresyl violet counterstained) in aged control motor cortex. Betz cells (open arrows) are rich in Nissl substance (blue, cresyl violet counterstaining) and have low BRCA1 immunoreactivity (brown). **d** Immunohistochemical staining for BRCA1 in ALS motor cortex. Betz cells (open arrows) are enriched in BRCA1 immunoreactivity (brown, cresyl violet counterstaining). **e** In ALS motor cortex layer five pre-attritional pyramidal neurons with distinct Nissl bodies (blue, cresyl violet counterstaining), BRCA1 immunoreactivity (brown) was localized in large cytoplasmic inclusions and was present only faintly in the nucleus (asterisk). **f** In layer five attritional pyramidal neurons in ALS motor cortex, Nissl substance was dispersed and attenuated (blue, cresyl violet counterstaining) and BRCA1 immunoreactivity (brown) was enriched in the nucleus (asterisk) and cytoplasm. **g** In aged control spinal cord dorsolateral funiculus corticospinal tract, BRCA1 immunoreactivity (brown) was very sparse. **h** In ALS spinal cord corticospinal tract (laterodorsal funiculus), numerous large axonal swellings (hatched arrows) positive for BRCA1 were present. Inset shows a BRCA1-positive axonal swelling (hatched arrow) and a negative profile (solid arrow). Scale bars (in μm) = 60 (A, same for B), 8 (E), 4 (F), 5 (G, same for H), 1.5 (H inset)

Human BRCA1 is a susceptibility gene for breast and ovarian cancers [86] that functions as a tumor suppressor protein responsible for mediating signal transduction in DDR and DNA repair and for destroying cells if repair is unsuccessful [33]. Because c-Abl is abnormal in human ALS (Fig. 4), we investigated other iconic proteins involved in human cancers that might also be aberrant in human ALS brain and spinal cord. We screened many commercial antibodies to BRCA1 for specificity using cultured human neural cells and gene-specific knockdown of BRCA1 (Fig. 5a). A specific BRCA1 immunoreactive band was detected at ~ 220 kDa (Fig. 5a). Western blotting for BRCA1 in human motor cortex from age-matched controls and ALS cases revealed low levels of BRCA1 in control brain, but significantly higher (*p* < 0.01) levels of BRCA1 in ALS (Fig. 5b). Similarly, immunohistochemistry for BRCA1 showed low immunoreactivity in aged human control motor cortex (Fig. 5c) and spinal cord (Fig. 5g), but in human ALS motor cortex (Fig. 5d-f) and spinal cord (Fig. 5h) BRCA1 immunoreactivity was prominent. The cellular localization of BRCA1 in human ALS motor cortical neurons, including Betz cells, was striking. Non-attritional and pre-attritional neurons contained large BRCA1-positive cytoplasmic inclusions and sparse nuclear immunoreactivity (Fig. 5e). Other pyramidal neurons at attritional stages of degeneration were enriched impressively with both cytoplasmic and nuclear immunoreactivity for BRCA1 (Fig. 5f). Consistent with the presence of upper motor neuron degeneration in ALS, there was marked accumulation of BRCA1-positive axonal swellings in the spinal cord corticospinal tract (laterodorsal funiculus) of ALS cases (Fig. 5h) that were not evident or very infrequent in age-matched control corticospinal tract axons (Fig. 5g).

### OHdG immunoreactivity is increased in vulnerable neurons and is also present in glia

**Fig. 6:**
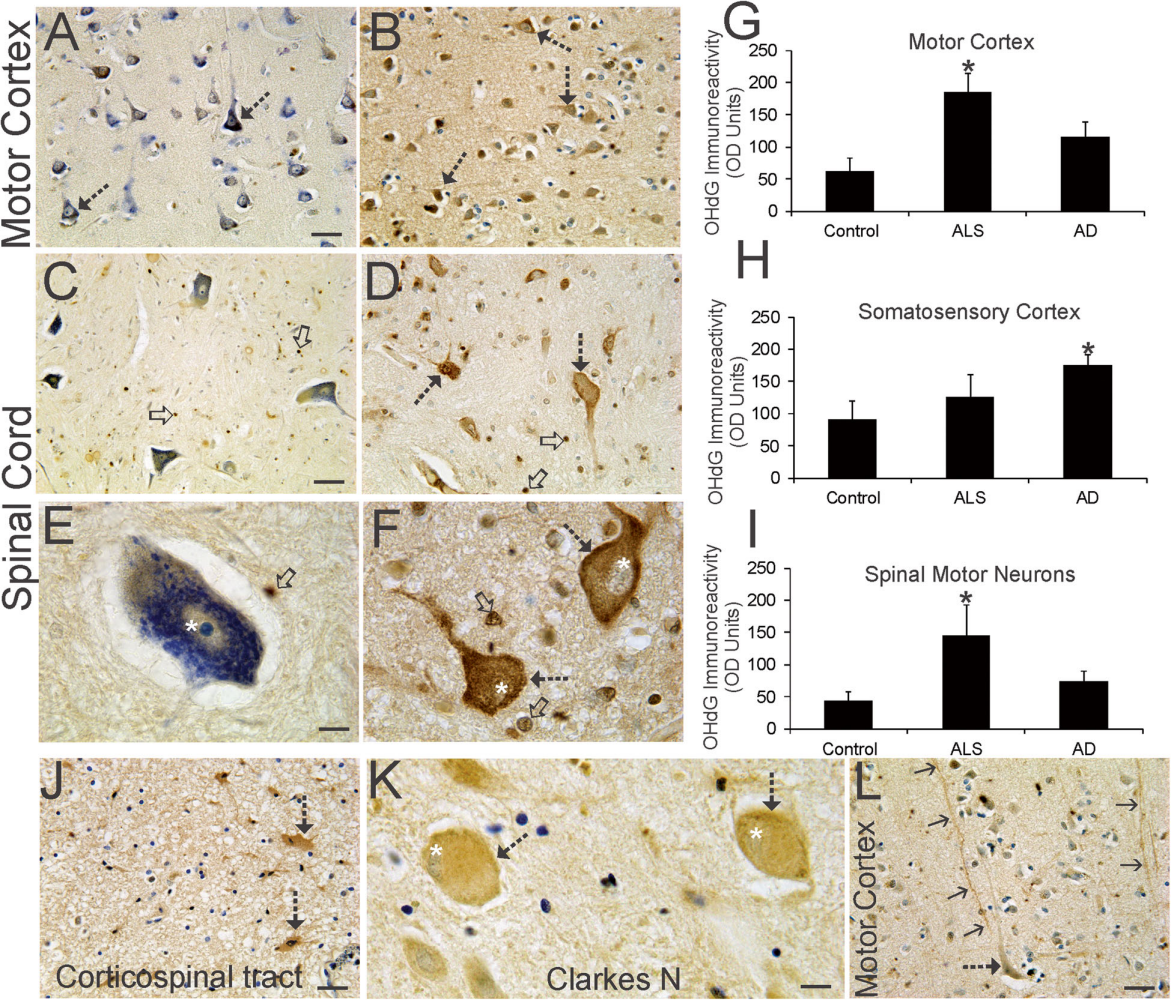
OHdG Immunoreactivity Accumulates in ALS Upper and Lower Motor Neurons and in Glia. **a** Immunohistochemical staining for OHdG (brown) with cresyl violet counterstaining in aged control motor Kim et al. 29 cortex. Large and small pyramidal neurons (open arrows) have low OHdG immunoreactivity in the nucleus. OHdG immunoreactivity in the neuropil was low. **b** In ALS motor cortex, many neurons (hatched arrows) were strongly positive for OHdG immunoreactivity (brown, cresyl violet counterstaining). Neuropil OHdG immunoreactivity is augmented compared to control (A). **c** In aged control ventral horn of spinal cord, motor neurons had prominent Nissl staining (blue, cresyl violet counterstaining) and low OHdG immunoreactivity (brown). Small glial cells showed strong nuclear OHdG immunoreactivity (open arrows, brown). **d** In ALS spinal cord, ventral horn motor neurons (hatched arrows) were strongly positive for OHdG (brown) as were small glial cells (open arrows). **e** Aged control spinal motor neurons were large and rich in Nissl substance and had diffusely distributed cytoplasmic OHdG immunoreactivity and modest OHdG immunoreactivity in the nucleus possessing a prominent nucleolus. A nearby glial cell (arrow) was intensely positive for OHdG. **f** ALS spinal motor neurons were attritional (hatched arrows) with severely dissipated Nissl substance and were enriched in OHdG immunoreactivity in the cytoplasm and nucleus (white asterisks). Many glial cells (open arrows) showed strong OHdG positivity. **g**-**i** Single-cell densitometry [111] of OHdG immunoreactivity in pyramidal neurons in motor cortex (G) and primary somatosensory cortex (H) and in ventral horn motor neurons of lumbar and cervical spinal cord of individuals with ALS (*n* = 16) and AD (*n* = 10) and aged matched non-neurological disease controls (*n* = 8). Values are mean ± SD. *p < 0.01. **j.** Large reactive astrocytes (hatched arrows) in the spinal cord corticospinal tract in ALS cases were strongly positive for OHdG (brown, cresyl violet counterstain). **k** Large neurons in Clarke’s nucleus (hatched arrows) displayed OHdG immunoreactivity (brown, cresyl violet counterstain) intermediate between control spinal motor neurons (E) and ALS spinal motor neurons (F), though they did appear chromatolytic. Their eccentrically positioned nucleus (white asterisks) had faint OHdG immunoreactivity. **l** In ALS motor cortex, some lengthy apical dendrites (arrows) of layer five pyramidal neurons were strongly positive for OHdG immunoreactivity. Scale bars (in μm) = 45 (A, same for B), 48 (C, same for D), 12 (E, same for F), 48 (J), 14 (K), 48 (L)

Because there are forms of DNA damage distinct from DNA strand-breaks [52, 66] and often-studied independent from the DDR, we assessed OHdG to expand on the identification of lesions in ALS motor neurons that potentially threaten their genomic integrity. OHdG is a DNA damage marker that detects oxidative damage as deoxyguanosine and is a footprint for free radical attack on DNA [27]. OHdG immunoreactivity is present in human control brain and spinal cord (Fig. 6a, c, e, g-i) and is seen in subsets of neurons and glia at least as low signal in the cytoplasm and nucleus, consistent with other work [47]. Some macroglia in control spinal cord had intense nuclear labeling (Fig. 6c, d). Because of the constitutive level of OHdG immunoreactivity, single individualized cell densitometry [73, 111] was used to assess the level of immunoreactivity, rather than counting discernable positive cells compared to negative cells. Moreover, because changes in OHdG levels may lack neurological disease specificity or may not signify disease or injury in cells [59, 93], we included assessments of OHdG immunoreactivity in AD brain. In ALS motor cortex, layers III-V pyramidal neurons showed significantly elevated OHdG immunoreactivity compared to age-matched controls and AD (Fig. 6a, b, g). Some layer V Betz pyramidal neurons displayed prominent dendritic immunoreactivity for OHdG (Fig. 6l). In contrast, OHdG was elevated compared to aged-controls in somatosensory cortex in AD but not in ALS (Fig. 6h). In ALS spinal cord, motor neurons had strong OHdG immunoreactivity in the cytoplasm, often obliterating the Nissl substance evident in age-matched control motor neurons, and in the nucleus (Fig. 6c-f). The level of OHdG immunoreactivity was significantly elevated in spinal motor neurons in ALS cases (Fig. 6i), but in other regions of spinal cord such as Clarke’s nucleus (Fig. 6k), origin of the dorsal spinocerebellar tract [14], OHdG immunoreactivity was lesser compared to motor neurons. However, strong OHdG immunoreactivity was not exclusive to neurons because many glial cells in ALS motor cortex and spinal cord, including the corticospinal tract, had intense positivity (Fig. 6b, d, f, j). Controls also had OHdG-imminoreactive glia (Fig. 6a, c, e). ELISA assay confirmed the elevations in OHdG immunoreactivity in ALS motor cortex and spinal cord ventral horn compared to age-matched controls (Table 6).

**Table 6:** 8-OHdG Levels in Human Control and ALS CNS Regions^a^

CNS Region	8-OHdG Concentration (pg/μl, mean ± SD)	
	Control	ALS
Motor Cortex	24 ± 11	35 ± 15*
Somatosensory Cortex	32 ± 14	29 ± 12
Spinal Cord-ventral horn	17 ± 7	27 ± 13*
Spinal Cord-dorsal horn	15 ± 11	18 ± 10

^a^8-OHdG levels were measured by ELISA. Tissue samples were obtained

by careful anatomical micropunch technique

*p < 0.05

### Accumulation of OHdG immunoreactivity in ALS motor neurons associates with cell death markers

**Fig. 7:**
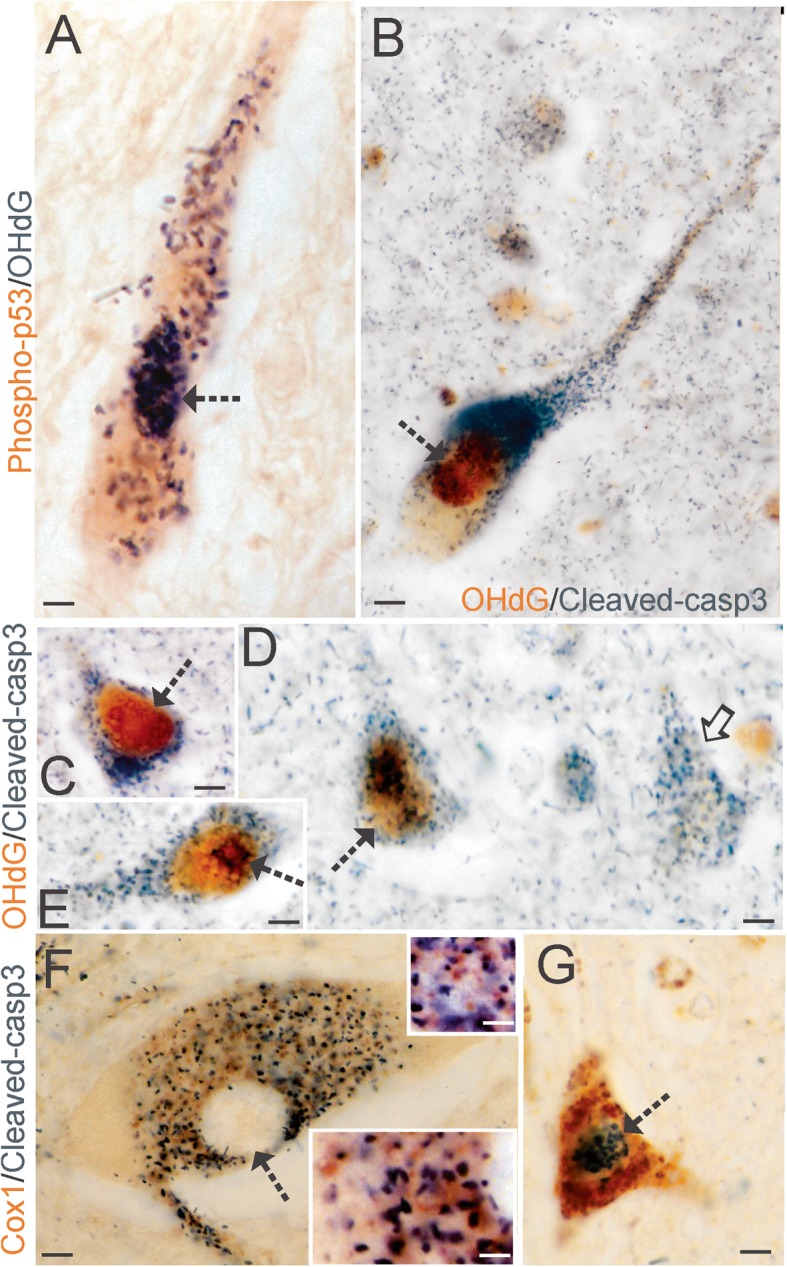
DNA Damage Coincides with Cell Death Markers in ALS Motor Neurons. **a** Spinal motor neurons in ALS showed colocalization of activated p53 (brown) and accumulated OHdG (black/dark green). Dual antigen labeling was done using DAB (brown) and BDHC (black/dark green) as chromogens [30] to avoid the pitfalls of immunofluorescence in aged postmortem human CNS tissues [45]. Colocalization was present in the nucleus Kim et al. 30 (hatched arrow) and in cytoplasmic particles. **b** Spinal motor neurons in ALS showed colocalization of accumulated OHdG (brown) and cleaved caspase-3 (black/dark green). **c-d** Cortical pyramidal neurons showed colocalization of accumulated OHdG (brown) and cleaved caspase-3 (black/dark green) and different neurons showed OHdG immunoreactivity in nuclear subdomains. In some neurons (C, hatched arrow), the nuclear OHdG immunoreactivity was mostly homogenous, but in other neurons (E, D) nuclear OHdG immunoreactivity was seen as granular particles (D, hatched arrow) and perinucleolar decorations (E, hatched arrow). Some cortical pyramidal neurons showed cleaved caspase-3 immunoreactivity but not OHdG immunoreactivity (D, solid arrow). **f** Spinal motor neurons in the chromatolytic pre-attritional stage of degeneration [63] in ALS cases showed perikaryal cytoplasmic enrichment of cleaved caspase-3 (black/dark green) and mitochondria (brown), identified by cytochrome c oxidase subunit 1 (Cox1) immunoreactivity, but the eccentrically placed nucleus was devoid of cleaved caspase-3 positivity (hatched arrow). Insets: different cytoplasmic regions where cleaved caspase-3 (black/dark green) is in association with discrete mitochondria (brown). **g** Spinal motor neurons (hatched arrow) in the attritional stage of degeneration [63] in ALS cases showed nuclear enrichment of cleaved caspase-3 (black/dark green) and cytoplasmic accumulation of mitochondria (brown). Scale bars (in μm) = 33 (A), 20 (B), 12 (C-D), 7 (F) 3 (F inset top), 2.5 (F inset bottom), 8 (G)

To contextualize cellular OHdG positivity relative to a degenerative or cell death phenotype in ALS neurons, we did dual antigen labeling using immunoperoxidase with DAB and BDHC [30, 56, 70]. This approach circumvents the serious problem of autofluorescence in older human postmortem tissues [45]. Spinal motor neurons in somatodendritic attritional stages of degeneration [63] that were positive for OHdG were also positive for phospho-p53 (Fig. 7a) and cleaved caspase-3 (Fig. 7b). Similarly, OHdG and cleaved caspase-3 colocalized in pyramidal neurons in ALS motor cortex (Fig. 7c-e), but some cleaved caspase-3^+^ pyramidal neurons were not OHdG^+^ (Fig. 7d). Nuclear OHdG immunoreactivity in ALS cortical pyramidal neurons appeared as diffuse labeling throughout the nucleus and as focal compartmental labeling often decorating the nucleolus and nuclear membrane (Fig. 7d, e). A spatial positioning of OHdG within genomic DNA subcompartments has been described [140]. Cleaved caspase-3 in ALS motor neurons also showed noteworthy relationships to mitochondria. In spinal motor neurons at chromatolytic stages of degeneration, evinced by the eccentrically placed nucleus [63], cleaved caspase-3 immunoreactivity was cytoplasmic, but not nuclear, and associated in complexes with mitochondria (Fig. 7f). In spinal motor neurons at attritional stages of degeneration [63], cleaved capsase-3 was primarily nuclear and not complexed with mitochondria in the cytoplasm (Fig. 7g) suggesting a commitment to cell death [53].

### DNA repair genes are hypomethylated in ALS CNS

**Fig. 8:**
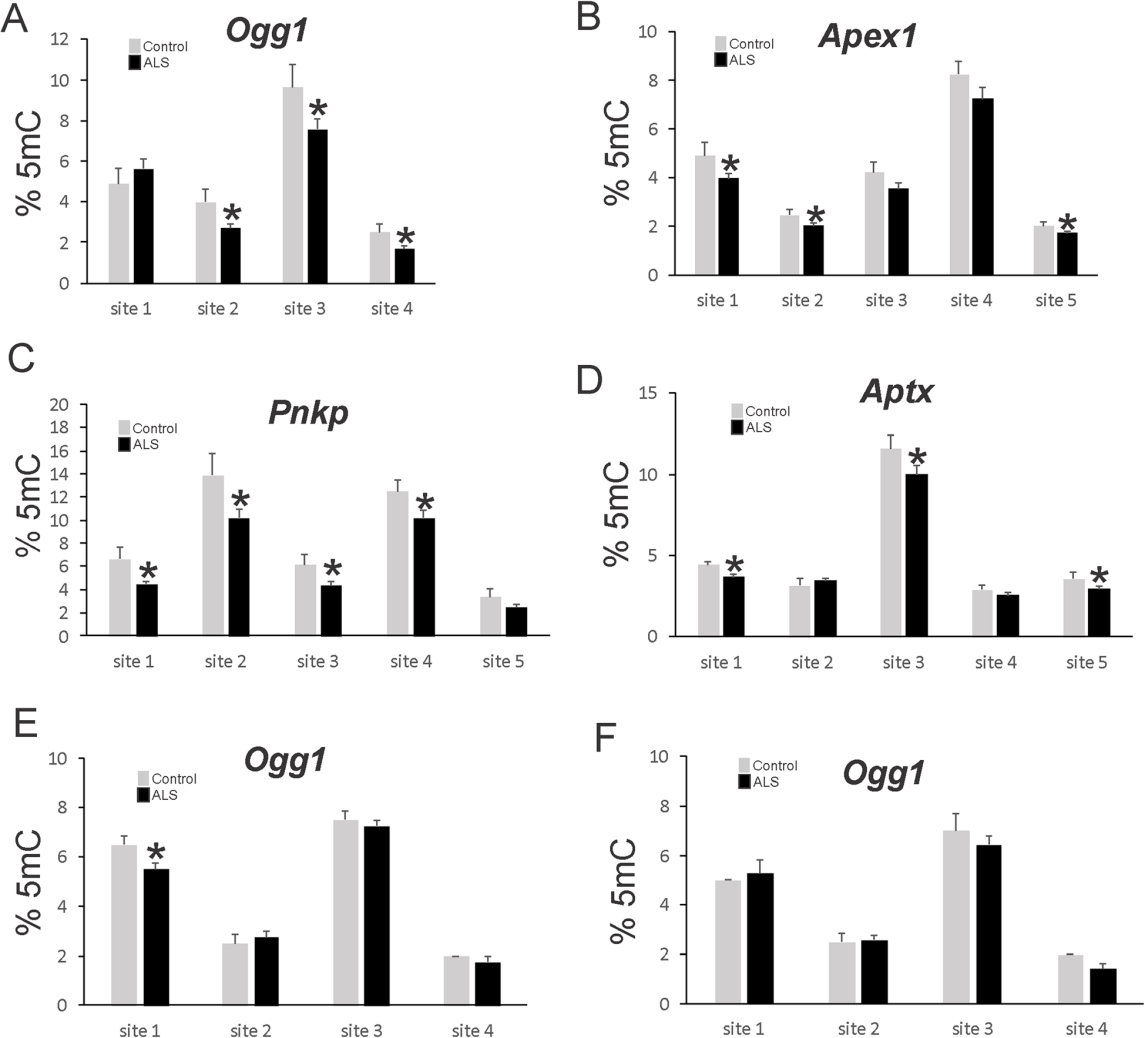
Gene-Specific Promoter DNA Methylation Pyrosequencing Reveals Hypomethylation of DNA Repair Genes in ALS. **a** 5-Methylcytosine (5mC) levels at four CpG sites in the *Ogg1* promoter in motor cortex of ALS and aged-matched control individuals. Values are mean ± SD. *p < 0.001. **b** 5mC levels at five sites in the *Apex1* promoter in motor cortex of ALS and aged-matched control individuals. Values are mean ± SD. *p < 0.01. **c** 5mC levels at five sites in the *Pnkp* promoter in motor cortex of ALS and aged-matched control individuals. Values are mean ± SD. *p < 0.01. **d** 5mC levels at five sites in the *Aptx* promoter in motor cortex of ALS and aged-matched control individuals. Values are mean ± SD. *p < 0.05. **e** 5mC levels at four sites in the *Ogg1* promoter in LCM-acquired spinal motor neurons of ALS and aged-matched control individuals. Values are mean ± SD. *p < 0.01. **f** 5mC levels at four sites in the *Ogg1* promoter in spinal cord dorsal horn of ALS and aged-matched control individuals. For A-F, *N* = 14 (ALS) and 8 (control)

To support the importance of DNA damage accumulation and DDR as possible pathological events in ALS we examined if there was evidence for epigenetic abnormalities. To this end, we used targeted gene promoter DNA methylation pyrosequencing to examine the epigenetic status of base excision repair and DNA single-strand break repair. In motor cortex, the *Ogg1* gene promoter showed significant demethylation of 3 of 4 CpG island sites in ALS cases compared to age-matched control (Fig. 8a). Western blotting confirmed the upregulation of OGG1 protein levels in ALS motor cortex compared to control (Additional file 2: Figure S2). Motor cortex in ALS also show significant CpG island demethylation compared to control at 2 of 5 sites in the *Apex1* gene (Fig. 8b), 4 of 5 sites in the *Pnkp* gene (Fig. 8c) and 2 of 5 sites in the *Aptx* gene (Fig. 8d). Specifically in spinal cord motor neurons, the *Ogg1* gene promoter showed significant demethylation of 1 of 4 CpG island sites in ALS cases compared to age-matched control (Fig. 8e), but no significant changes in *Ogg1* promoter methylation were seen in ALS dorsal horn Rexed laminae II, III, and IV (Fig. 8f).

### Human ALS motor neurons have capacity for DNA damage repair

**Fig. 9:**
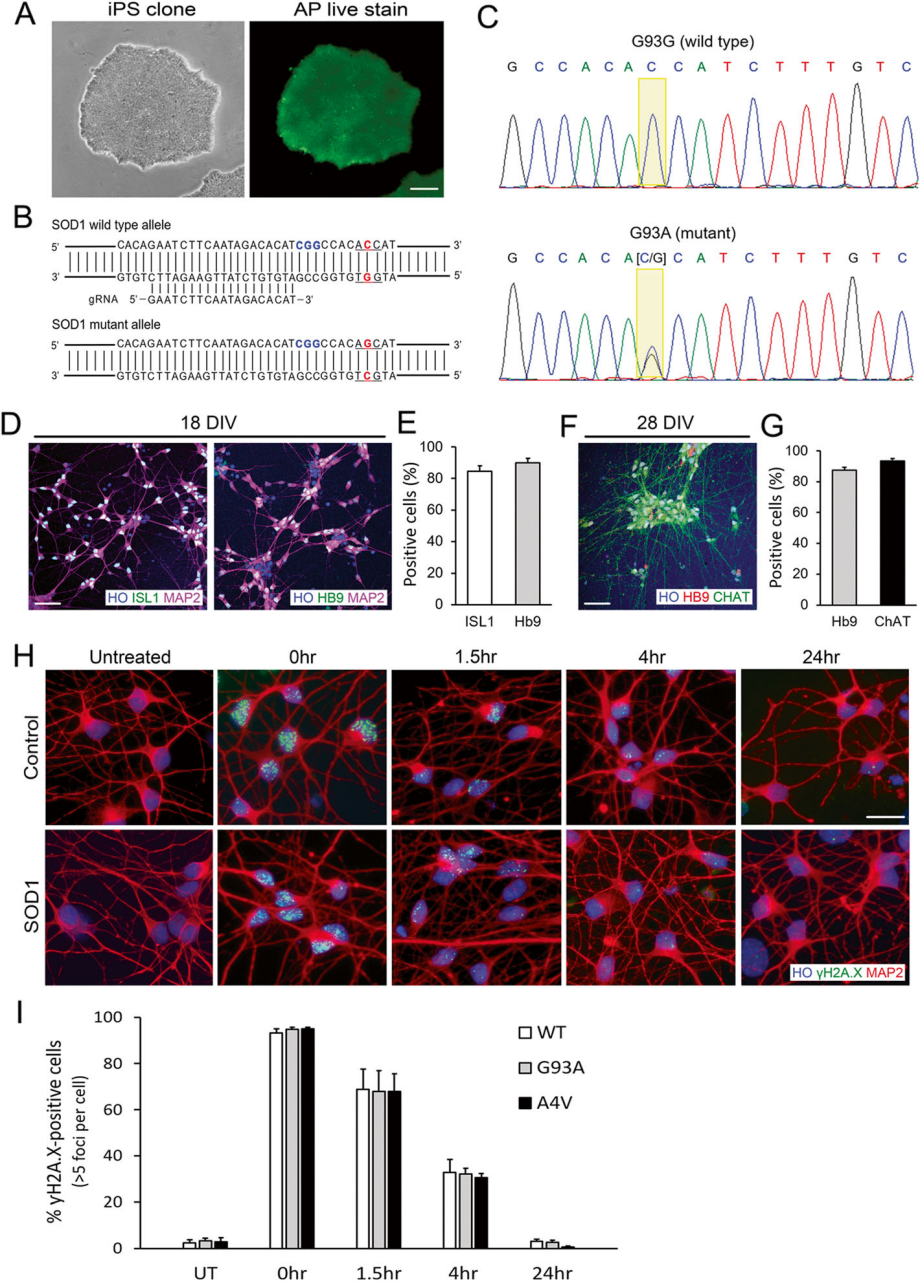
Human iPSC-derived Motor Neurons with SOD1 Mutations Show Capacity for DNA Repair. **a** Phase-contrast image of human iPSCs. **b** Alkaline phosphatase live staining showed stem cell pluripotency. **c** Guide RNA design targeting SOD1 wild type allele (**d**) Chromatogram showing CRISPR-Cas9 mediated genome editing of SOD1+/+ to SOD1+/G93A. **e**, **g** Immunofluorescence images and quantification of ISL1 and Hb9-positive motor neurons at Day 18. **f**, **h** Immunofluorescence images and quantification of Hb9 and ChAT-positive motor neurons at Day 28. **i**, **j** ƴH2A.X foci in iPSC-derived motor neurons after etoposide treatment. **K.** Quantification of ƴH2A.X foci at different recovery time points. Values are mean ± SD. Scale bars = 50 μm

Because ALS motor neurons showed significant DNA damage accumulation, DDR, and promoter hypomethylation in DNA repair genes in human postmortem CNS tissues (Figs. 1, 2, 3, 4, 5, 6, 7 and 8), and realizing that these are all static assessments, we investigated DNA repair capacity in living human iPSC-derived motor neurons (Fig. 9). The human iPSC lines used to derive motor neurons were a healthy control iPSC line (C3-1) [133] and two fALS-iPSC lines (Table 3, Fig. 9a). The fALS iPSC lines were from a patient carrying a SOD1-A4V mutation (GO013) [51], and one with a SOD1-G93A mutation that was generated by CRISPR-Cas9 genome editing. The isogenic non-mutated iPSC line of this latter cell line was an additional control (Table 3). To generate an isogenic iPSC line with a SOD1-G93A missense mutation, iPSC pluripotency was verified by alkaline phosphatase staining (Fig. 9a). A guide RNA that specifically targets the wild-type allele (Fig. 9b) and a single-stranded donor oligonucleotide were designed (Table 4), and together with Cas9 protein, they were delivered by electroporation into the cells to mediate genome editing. Single clones were isolated and the heterozygous SOD1-G93A mutation was confirmed by PCR amplification of the targeted region followed by direct DNA sequencing (Fig. 9c).

Using this genome-edited SOD1-G93A iPSC line along with its isogenic control wild-type and patient-derived SOD1-A4V iPSC lines, we differentiated cells into highly pure spinal motor neurons, as confirmed by motor neuron markers. At 18–21 days of differentiation, greater than 80% of the cells were ISL1 and Hb9 positive (Fig. 9d, e). Approximately 80–90% of the cells were positive for ChAT, a mature motor neuron marker, at 28–31 days of differentiation (Fig. 9f, g).

To assess directly DNA damage formation and DNA repair in living human control and ALS motor neurons, we treated the iPSC-derived motor neurons with etoposide and visualized DNA damage accumulation by γH2A.X immunoreactivity, a serine-139 phosphorylated form of H2A and an established marker for DNA damage, including double-strand breaks [103, 110] distinguished from DNA single-strand breaks [57]. We counted γH2A.X-positive foci in motor neuron nuclei to assess indirectly DNA repair capacity at several different recovery time points. γH2A.X immunoreactivity in untreated control and ALS motor neurons was low (Fig. 9h, i) suggesting that steady-state repair of baseline endogenous DNA double-strand breaks in ALS motor neurons is similar to control at 30 days of culture. After 1 h of 10 μM etoposide exposure, nearly all motor neurons in control and ALS cultures accumulated similar levels of DNA damage as seen by the accumulation of γH2A.X foci (Fig. 9h, i), suggesting that etoposide trapping of topoisomerase II and DNA-strand cleavage in ALS and control motor neurons were similar. Repair of DNA damage as seen by disappearance of γH2A.X foci was examined carefully over time and the number of foci per cell was quantified and compared at each time point. The numbers of γH2A.X foci in the SOD1 mutants decreased over time and were similar to wild type control at all recovery periods (Fig. 9h, i), demonstrating that iPSC-derived motor neurons with SOD1 mutations responded to DNA damage and repaired DNA damage with kinetics similar to control motor neurons.

## Discussion

Our study shows that motor neurons in human ALS accumulate DNA damage and have the capacity to respond to DNA damage by activating DDR sensor effectors, recruitment of proteins to the nucleus, and epigenetic hypomethylation of DNA repair genes. We identified directly three forms of DNA damage [52, 66] accumulating in diseased human motor neurons in vivo: AP sites, single-stranded DNA, and OHdG. The DDR sensors that appear activated consequently are c-Abl, ATM, BRCA1, and p53. We also identified hypomethylation of several DNA repair genes in the CNS of individuals with ALS, supporting a previous study showing upregulation and activation of APE1 in human ALS brain [111]. Experimental results on postmortem human ALS tissue unveil static events at endstage disease and are non-dynamic; therefore, we also studied living human ALS and control motor neurons generated by iPSC/genome-editing in cell culture. After characterizing our cell culture model, experiments on human SOD1 mutant iPSC-derived motor neurons revealed that DDR was activatable in diseased motor neurons, as evidenced by the accumulation of phosphorylated H2A.X, and that DNA repair capacity and kinetics in ALS motor neurons were similar to wildtype motor neurons, as reported by the disappearance of phosphorylated H2A.X. Thus, DNA damage accumulation is a major phenotype of human motor neuron degeneration in ALS that is associated with significant epigenetic and post-translational DDRs that are mobilized and recruited to the nucleus in human ALS motor neurons in vivo and that DDR and DNA repair are engaged and functional in human mutant SOD1 ALS motor neurons in cell culture.

### DNA damage accumulation in human motor neurons

DNA damage is defined as any modification of DNA that changes its coding properties or normal function in transcription or replication [52, 101]. DNA lesions can occur in many different forms, including apurinic/apyrimidinic (AP) sites (abasic sites), adducts, single-strand breaks, double-strand breaks, DNA-protein crosslinks, and insertion/deletion mismatches [52, 101]. We found in human ALS significant accumulation of AP sites in vulnerable brain regions and, specifically, in spinal motor neurons. These DNA lesions are formed either spontaneously by free radicals or as intermediates during the course of normal repair of oxidized, deaminated, or alkylated bases [2, 52]. AP sites are a major type of damage generated by reactive oxygen species (ROS). Estimates indicate that endogenous ROS can cause approximately 50,000–200,000 AP sites per day in the genome of mammalian cells, and that brain cells contain high AP sites [2]. Aberrant redox chemistry and oxidative stress is a leading putative mechanism of pathogenesis in ALS [5]. Edaravone, an antioxidant that protects neurons in vivo [94, 117], is approved by the FDA for the treatment of ALS [132], though its efficacy and mechanisms of action need evaluation. This drug can attenuate neuronal nuclear DNA damage caused by nitrative stress and hydroxyl radical (·OH) in brain in vivo [94, 117]. Edavarone also appears to stimulate DNA repair [11, 38, 116]. Thus, enforced DNA damage repair in motor neurons could mediate the clinical efficacy of edaravone in ALS patients, as DNA repair enforcement rescues motor neurons in mice [83].

The detection of single-stranded DNA with monoclonal antibody F7-26 allows for discrimination between apoptosis and necrosis in many cell types [28, 29, 99]. We localized ssDNA specifically in the nucleus of upper and lower motor neurons, while non-motor neurons in the same tissue section (post-central gyrus of cerebral cortex and spinal cord dorsal horn) were unlabeled. In animal and cell models and in human neurons, we have found consistently that ssDNA is formed early in the progression of neurodegeneration (both apoptotic and hybrid forms) in vivo and in cell culture [54, 55, 76–80]. The timing for ssDNA accumulation in our models of neurodegeneration, and as shown here in pre-attritional and attritional motor neurons in human ALS, places them as possible upstream activators of a p53-dependent neuronal cell death process [76, 79].

OHdG is an oxidized DNA base lesion [27]. We found elevations in OHdG in human ALS motor regions and specifically in LCM-acquired motor neurons. Surprisingly, we did not find elevated levels of OHdG in ALS mitochondrial DNA. Previously, OHdG was found elevated in postmortem CNS extracts of individual with ALS [26]. OHdG can be generated from ·OH [112]. ^∙^OH can be formed by either the Fenton reaction involving homolytic cleavage of hydrogen peroxide catalyzed by Fe^2+^ or Zn^2+^, the latter possibly released from SOD1 [125], or by the decomposition of peroxynitrite (ONOO^-^) that is formed by the combination of superoxide and nitric oxide [4]. ONOO^-^ has prominence in the mechanisms of pathogenesis in ALS [5], and ONOO^-^ induces several forms of genomic DNA damage directly in rodent motor neurons [54, 55]. Specific loci in DNA sequences can accumulate OHdG [121, 140], and DNA damage might accumulate preferentially in some promoter regions in the genome of the aging human brain [59]. These possibilities are relevant to ALS because we observed nuclear subcompartmentation in the accumulation of OHdG with peri-nucleolar and peri-nuclear envelope DNA damage occurring seemingly before large-scale and generalized chromatin damage in ALS motor neurons. In cell-free biochemical systems, the amount of OHdG relates linearly to the levels of DNA single-strand breaks [122]. In rodents, we have found the accumulation of OHdG lesions in pre-apoptotic neurons during dying-back retrograde degeneration [1, 73, 74, 81], a process implicated in the pathobiology of ALS [25, 78, 138], and in pre-necrotic neurons during ischemic neurodegeneration [71]. Thus, it is unlikely that the accumulation of genomic OHdG footprints any particular form of cell death, though in human ALS motor neurons, we find definitive coincidence of OHdG with phosphorylated p53 that is an iconic driver of apoptosis and cellular senescence [36] and is strongly upregulated in human ALS [64].

Nuclear abnormalities in motor neurons have been implicated in the pathogenesis of human ALS for a long time. DNA damage accumulation, DNA repair dysfunction, and RNA defects have all been described [7, 20, 21, 61, 65, 78, 102, 108]. Much of this earlier work lacked details regarding molecular mechanisms, including forms of DNA damage and types of DDR; however, the concept is now substantiated by this work and other studies of familial ALS mutant genes in cell culture, including *SOD1*, *C9orf72*, *fused in sarcoma* (*FUS*), and *TAR DNA-binding protein 43* (*TDP43*) [23, 41, 87]. Moreover, wildtype FUS and TDP43 proteins have been found to localize to sites of DNA damage in human osteosarcoma epithelial cells and appear to function in the prevention of transcription-coupled DNA damage and in repair of DNA repair [42].

### The DDR is activated in ALS

We found changes in several proteins that function in DNA damage sensing in human ALS CNS compared to age-matched controls, thus confirming that DNA damage is present in diseased motor neurons. Phosphorylated c-Abl was strongly elevated in the nucleus and cytoplasm of vulnerable upper and lower motor neurons. Some spinal motor neurons highly enriched in activated c-Abl appeared in the degenerative stage of chromatolysis or pre-attrition [63]. Western blotting confirmed the upregulation of phosphorylated and total c-Abl in ALS brain. c-Abl, a non-receptor protein tyrosine kinase possessing nuclear localization and nuclear export signals, shuttles between the nucleus and cytoplasm [115]. c-Abl binds chromatin is activated by ATM, and, in turn, functions in amplifying ATM activation, and modulating cellular responses to DNA double-strand breaks [60, 129]. The accumulation of c-Abl in the nucleus of motor neurons is meaningful because it suggests involvement in the mechanisms of motor neuron degeneration in ALS and persistence of functional nuclear import in ALS motor neurons. Our findings on c-Abl in postmortem human CNS of ALS cases are also significant because of recent indications that c-Abl is a potential therapeutic target for ALS, identified in studies of human ALS patient iPSC-derived motor neurons [45]. Consistent with the phosphorylation of c-Abl is evidence of ATM activation, gleaned from ATM phosphorylated targets. Postmortem assessment of C9orf72 mutated ALS cases has revealed activation of ATM and other evidence for induction of DDR [23]. Phosphorylation of ATM target proteins coincides with accumulation of phosphorylated ATM in cortical neurons undergoing DNA damage-induced apoptosis [79]. Inhibition of c-Abl kinase with the small molecule STI571 (Gleevec, Imatinab mesylate), used clinically to treat some forms of leukemia, myelodysplasia, and gastrointestinal cancers, blocked cortical neuron apoptosis [79], thereby supporting the interpretation of c-Abl activation in human motor neurons in ALS is mechanistically relevant, and consistent with the idea that c-Abl inhibition could be relevant therapeutically in ALS [45].

Another indicator of DNA damage presence in ALS motor neurons was upregulation and cellular accumulation of BRCA1 protein as seen by immunohistochemistry and western blotting. BRCA1 is required for transcription-coupled repair of oxidatively damaged DNA [126] and functions in homologous recombination repair of DNA double-strand breaks [92] through its association with ATM [18] and other partners [33]. Interestingly, FUS and TDP43 proteins can collaborate with BRCA1 in repair of transcription-coupled DNA damage [42]. The abundance of BRCA1 is cell cycle regulated and increased as cells enter S phase and is low in G_o_ and G_1_ cells [12]. In human motor neurons, BRCA1 immunoreactivity was scarce in control cells, consistent with the postmitotic state of mature neurons, but BRCA1 was present ubiquitously in ALS motor neurons. In ALS neurons without morphological evidence of attrition, BRCA1 was found compartmentalized as large cytoplasmic clumped and granular inclusions, but immunoreactivity was low in the nucleus. Some of the cytoplasmic BRCA1 immunoreactivity could be mitochondrial [15]. In contrast, in attritional ALS neurons, BRCA1 was enriched throughout the cell and was prominent in the nucleus, putatively marking the presence of DNA damage and further identifying operative nuclear import mechanisms. Alternatively, our data confirm that diseased ALS motor neurons re-enter S-phase of the cell cycle, as suggested in assessments of human ALS postmortem tissue [100] and in cell and mouse models of ALS [130].

The study of human postmortem CNS yields static data that is often interpreted as temporal sequences of events regarding mechanisms of disease, but this is not a true dynamic representation of disease. We therefore developed, characterized, and then employed a human iPSC-derived motor neuron cell culture model to study DNA damage and DNA repair in living ALS and control motor neurons. Familial SOD1 mutant motor neurons were prepared from patient-derived iPSCs and CRISPR/Cas9 genome-edited iPSCs using modifications of existing protocols for directed differentiation of iPSCs based on principals of embryogenesis and neurodevelopment [9, 22, 84]. Our differentiated human motor neurons met the standard definition of motor neurons based on size and multipolar morphology and immunophentyping for ISL1, HB9, and ChAT positivity. We used etoposide to generate DNA double-strand brakes and γH2A.X immunoreactivity as a reporter for the accumulation of DNA strand breaks and their subsequent repair by the disappearance of γH2A.X foci. This experiment showed that DNA damage accumulation and repair of DNA double-strand breaks in ALS and control motor neurons were similar. However, this experiment does not show that DNA repair in ALS motor neurons has fidelity. Sequencing experiments are needed to determine if DNA repair in ALS motor neurons is true and faithful or if it is error-prone.

### p53 activation and cleaved caspase-3 are found in human ALS motor neurons with DNA damage

p53 functions in DDR, growth control, and cell death in cycling cells [31], but in postmitotic mature adult CNS neurons, p53 functions are less well known, though neuronal apoptosis is a key function [73, 75, 76, 135]. We found that phospho^ser15^-activated p53 accumulates in the nucleus of human ALS motor neurons with DNA damage. p53 is activated by genotoxic stress and can trigger the onset of DNA repair or classical apoptosis [31, 134]. The intricacies and nuances of p53 involvement in DNA repair are manifold [134]. p53^-/-^ mice exhibit an increase in chromosomal abnormalities and deficiencies in global genomic DNA repair [31]. It could be the non-apoptotic cellular repair aspects of p53 that are relevant to our observations in human ALS motor neurons, where p53 is strongly activated, but there is no morphological evidence for classical neuronal apoptosis [63, 64, 67]. Nucleotide excision repair and base excision repair pathways can involve p53 through its ability to interact with components of the repair machinery. APE1 is a strong partner for p53 as a regulator of expression, stability, and function [134]. It is interesting that both APE1 and p53 are upregulated in human ALS motor neurons [64, 111]. Generally, p53 is a short-lived protein with a half-life of approximately 5–20 min in many different cell types [31]. p53 is regulated by posttranslational modifications (phosphorylation and acetylation) and is modulated by intracellular redox state [50]. Protein levels of p53 can rapidly increase several-fold after DNA damage, mainly by post-translational mechanisms. Phosphorylation of p53 at serine^15^ by ATM is a key response to DNA damage. We found evidence for activation of ATM in human ALS brain. The elevation in p53 protein levels occurs through stabilization stimulated by phosphorylation. ATM also regulates the stabilization of p53 through Mdm2 phosphorylation, thus preventing Mdm2-dependent p53 degradation. We have found previously that p53 is activated and has functional DNA-binding in human ALS CNS, as identified by electrophoretic mobility shift assay [64]. p53 also is enriched in the nucleus of attritional motor neurons in ALS [64]. Others have confirmed this finding [100]. Because numerous animal species differences exist concerning p53 function, notably between mouse and human [43], future studies using human iPSCs are needed to decipher the beneficial and reparative and the degenerative actions of p53 in human motor neurons.

Like our p53 observations, cleaved caspase-3 was found in cortical and spinal motor neurons with DNA damage in human ALS cases. Interestingly, cleaved caspase-3 was localized differently in motor neurons depending on the stage of degeneration. Chromatolytic pre-attritional motor neurons were enriched in cytoplasmic cleaved caspase-3 in apparent association with mitochondria. In contrast, in attritional motor neurons, the cleaved caspase-3 was segregated from mitochondria and was enriched in the nucleus. This finding is consistent with the proteolytic actions of cleaved caspace-3 on nuclear proteins during apoptosis [46] and with observations seen in human cell culture [46, 98]. However, injured or degenerating human neurons in vivo rarely show morphological evidence of classical apoptosis [63, 67, 95], and it is possible that the accumulation of cleaved caspase-3 in ALS motor neurons is independent of a stereotypic apoptotic process, but, rather is related to some non-apoptotic form of neuronal cell death falling along the cell death continuum [65, 67, 69, 95]. Alternatively, the nuclear cleaved caspase-3 is unrelated to cell death in general [88] and is participating in non-lethal activities in human ALS motor neurons.

### DNA methylation in human ALS

We interrogated DNA repair gene silencing by methylation as a mechanism of disease in ALS. However, gene-specific promoter DNA methylation pyrosequencing identified the DNA repair genes *Ogg1*, *Apex1*, *Pnkp* and *Aptx* as hypomethylated in ALS. Few papers report on DNA methylation in human ALS [8]. DNA methylation in sporadic ALS has been examined in disease candidate genes *SOD1, vascular endothelial growth factor, angiogenin,* and *TDP43* [8, 96], in members of the metallothione gene family [89], and in the glutamate transporter *EAAT2* gene promoter [139]. None of these studies found differences in methylation patterns in sporadic ALS cases compared to control cases. Studies of *C9orf72* promoter methylation in sporadic ALS yield contradictory results [8]. A genome-wide analysis of brain DNA methylation in sporadic ALS accomplished by chromatin immunoprecipitation followed by microarray hybridization revealed significant hypermethylation of genes involved in calcium dynamics, oxidative stress, and synapses [90]. However, most of the DNA methylation was found in non-promoter regions (intronic and cyrptic) and the brain tissue analyzed was the dorsolateral prefrontal cortex [90]. This region of cerebral cortex (Brodmann area 46) is non-motor and controls executive functions, including working memory and selective attention [106]. It is usually unaffected neuropathologically in ALS, unless there is dementia associated with the disease. In sporadic ALS spinal cord hypomethylation was identified in a variety of genes involved in inflammatory and immune responses [24]. A recent blood methylome analysis of monozygotic twins discordant for ALS identified the DDR gene RAD9B as differentially methylated [118], consistent with the activation of DDR and DNA repair shown here.

## Conclusions

We found in postmortem CNS tissue evidence for the accumulation of several different forms of DNA damage and engagement of a significant DDR in human ALS motor neurons demonstrated by activation and nuclear recruitment of DNA damage sensor proteins and DNA repair gene hypomethylation. These results are complemented by evidence that human ALS iPSC-derived motor neurons can engage a strong DDR with a repair capacity similar to wildtype motor neurons.

## Supplementary information

**Additional file 1: Figure S1.** Validation of mitochondrial DNA purification and DNA-AP site assay sensitivity. **A.** Agarose gel electrophoresis showing purified DNA isolated from mitochondria of human motor cortex. Band size at approximately 16 kb is consistent with mitochondrial, and the band is RNase insensitive but completely sensitive to DNase. **B.** Standard curve for the sensitivity of DNA-AP site number. The assay can detect fewer than five AP sites in 1 × 10^5^ bp DNA, and the detection is essentially linear.

**Additional file 2: Figure S2.** Western blot for OGG1 proteins levels in human ALS and control motor cortex. Positive control (^+^control) is human recombinant OGG1.

## Data Availability

Data will be shared at the request of Lee J. Martin (martinl@jhmi.edu).

## Abbreviations

5mC: 5-methylcytosine
ALS: Amyotrophic lateral sclerosis
AP site: Apurinic/apyrimidinic site (abasic site in DNA)
ATM: Ataxia telangiectasia mutated protein kinase
BRCA1: Breast cancer type 1 susceptibility protein
c-Abl: Abelson non-receptor tyrosine kinase
DDR: DNA damage response
fALS: Familial ALS
iPSC: induced pluripotent stem cell
LCM: Laser capture microdissection
OHdG: 8-hydroxy-deoxyguanosine
SOD1: Superoxide dismutase-1
ssDNA: single-stranded DNA

## References

[1] Al-Abdulla NA, Martin LJ (1998). Apoptosis of retrogradely degenerating neurons occurs in association with the accumulation of perikaryal mitochondria and oxidative damage to the nucleus. Am J Pathol.

[2] Atamna H, Cheung I, Ames BN (2000). A method for detecting abasic sites in living cells: age-dependent changes in base excision repair. Proc Natl Acad Sci.

[3] Barber SC, Shaw PL (2010). Oxidative stress in ALS: key role in motor neuron injury and therapeutic target. Free Rad Biol Med.

[4] Beckman JS, Carson M, Smith CD, Koppenol WH (1993). ALS, SOD and peroxynitrite. Nature.

[5] Beckman JS, Koppenol WH (1996). Nitric oxide, superoxide, and peroxynitrite: the good, the bad, and the ugly. Am J Physiol.

[6] Bogdanov M, Brown RH, Matson W, Smart R, Hayden D, O’Donnel H (2000). Increased oxidative damage to DNA in ALS patients. Free Rad Biol Med.

[7] Bradley WG, Krasin F (1982). A new hypothesis of the etiology of amyotrophic lateral sclerosis. The DNA hypothesis. Arch Neurol.

[8] Bruggeman EC, Yao B (2019) DNA methylation in neuronal development and disease. In: The DNA, RNA, and Histome Methylomes, Jurga J, Barciszewski (eds.), pp103-140, Springer Nature: Switzerland

[9] Calder EL, Tchieu J, Steinbeck JA (2015). Retinoic Acid-Mediated Regulation of GLI3 Enables Efficient Motoneuron Derivation from Human ESCs in the Absence of Extrinsic SHH Activation. J Neurosci.

[10] Chang Q, Martin LJ (2009). Glycinergic innervation of motoneurons is deficient in amyotrophic lateral sclerosis mice: a confocal quantitative analysis. Am J Pathol.

[11] Chen L, Liu Y, Dong L, Chu X (2015). Edaravone protects human peripheral blood lymphocytes from γ-irradiation-induced apoptosis and DNA damage. Cell Stress Chaperones.

[12] Chen Y, Farmer AA, Chen C-F, Jones DC, Chen P-L, Lee W-H (1996). BRCA1 is a 220-kDa nuclear phosphoprotein that iss expressed and phosphorylated in a cell cycle-dependent manner. Cancer Res.

[13] Chen YZ, Bennett CL, Huynh HM, Blair IP, Puls I, Irobi J (2004). DNA/RNA helicase gene mutations in a form of juvenile amyotrophic lateral sclerosis (ALS4). Am J Hum Genet.

[14] Clarke JL (1859). Further researches on the grey substance of the spinal cord. Philosophical Trans Royal Soc Lond.

[15] Coene ED, Hollinshead MS, Waeytens AA, Schelfhout VR, Eechaute WP, Shaw MK (2005). Phosphorylated BRCA1 is predominantly located in the nucleus and mitochondria. Mol Biol Cell.

[16] Coppede F, Mancuso M, Lo Gerfo A, Carlesi C, Piazza S, Rocchi A (2007). Association of the hOGG1 Ser326Cys polymorphism with sporadic amyotrophic lateral sclerosis. Neurosci Lett.

[17] Coppede F, Mancuso M, Lo Gerfo A, Manca ML, Petrozzi L, Migliore L (2007). A Ser326Cys polymorphism in the DNA repair gene hOGG1 is not associated with sporadic Alzheimer’s disease. Neurosci Lett.

[18] Cortez D, Wang Y, Qin J, Elledge SJ (1999). Requirement of ATM-dependent phosphorylation of Brac1 in the DNA damage response to double-strand breaks. Science.

[19] Cradick TJ, Qiu P, Lee CM (2014). COSMID: a Web-based tool for identifying and validating CRISPR/Cas off-target sites. Mol Ther Nucleic Acids.

[20] Davidson TJ, Hartman HA (1981). RNA content and volume of motor neurons in amyotrophic lateral sclerosis. J Neuropathol Exp Neurol.

[21] Davidson TJ, Hartman HA (1981). Base composition of RNA obtained from motor neurons in amyotrophic lateral sclerosis. J Neuropath Exp Neurol.

[22] Du ZW, Chen H, Liu H (2015). Generation and expansion of highly pure motor neuron progenitors from human pluripotent stem cells. Nat Commun.

[23] Farg MA, Konopka A, Soo KY, Ito D, Atkin JD (2017). The DNA damage response (DDR) is induced by C9orf72 repeat expansion in amyotrophic lateral sclerosis. Hum Mol Genet.

[24] Figueroa-Romero C, Hur J, Bender DE, Delaney CE, Cataldo MD (2012). Identification of epigenetically altered genes in sporadic amyotrophic lateral sclerosis PloS ONE.

[25] Fischer LR, Culver DG, Tennant P, Davis AA, Wang M, Castellano-Sanchez A (2004). Amyotrophic lateral sclerosis is a distalaxonopathy: evidence in mice and man. Exp Neurol.

[26] Fitzmaurice PS, Shaw IC, Kleiner HE, Miller RT, Monks TJ, Lau SS (1996). Evidence for DNA damage in amyotrophic lateral sclerosis. Muscle Nerve.

[27] Fraga CG, Shigenaga MK, Park J-W, Degan P, Ames BN (1990). Oxidative damage to DNA during aging:8-hydroxy-2-deoxyguanosine in rat organ DNA and urine. Proc Natl Acad Sci.

[28] Frankfurt OS (1990). Decreased stability of DNA in cells treated with alkylating agents. Exp Cell Res.

[29] Frankfurt OS, Robb JA, Sugarbaker EV, Villa L (1996). Monoclonal antibody to single-stranded DNA is a specific and sensitive cellular marker of apoptosis. Exp Cell Res.

[30] Furuta A, Price DL, Pardo CA, Troncoso JC, Xu Z, Taniguchi N, Martin LJ (1995). Localization of superoxide dismutases in Alzheimer's disease and Down's syndrome neocortex and hippocampus. Am J Pathol.

[31] Giaccia AJ, Kastan MB (1998). The complexity of p53 modulation: Emerging patterns from divergent signals. Genes Develop.

[32] Ginsberg SD, Hemby SE, Mufson EJ, Martin LJ, Zaborszky L, Wouterlood FG, Lanciego JL (2006). Cell and tissue microdissection in combination with genomic and proteomic profiling. Neuroanatomical Tract-Tracing 3. Molecules, Neurons, and Systems.

[33] Greenberg RA (2008). Recognition of DNA double strand breaks by the BRCA1 tumor suppressor network. Chromosoma.

[34] Guillet M, Boiteux S (2002). Endogenous DNA abasic sites cause cell death in the absence of Apn1, Apn2, and Rad1/Rad10 in Saccharomyces cerevisiae. EMBO J.

[35] Hamilton ML, Guo Z, Fuller CD, Van Remmen H, Ward WF, Austad SN (2001). A reliable assessment of 8-oxo-2-deoxyguanosine levels in nuclear and mitochondrial DNA using the sodium iodide method to isolate DNA. Nucleic Acid Res.

[36] Hansen R, Oren M (1997). p53: from inductive signal to cellular effect. Cur Opin Genet Devel.

[37] Harrison L, Brame KL, Geltz LE, Landry AM (2006). Closely opposed apurinic/apyrimidinic sites are converted to double strand breaks in Escherichia coli even in the absence of exonuclease III, endonuclease IV, nucleotide excision repair and AP lyase cleavage. DNA Repair.

[38] Hata K, Urushibara A, Yamashita S, Lin M, Muroya Y, Shikazono N (2015). Chemical repair activity of free radical scavenger edaravone: reduction of reactions with dGMP hydroxyl radical adducts and suppression of base lesions and AP sites on irradiated plasmid DNA. J Radiat Res.

[39] Hayward C, Colville S, Swingler RJ, Brock DJH (1999). Molecular genetic analysis of the APEX nuclease gene in amyotrophic lateral sclerosis. Neurology.

[40] Helbock HJ, Beckman KB, Ames BN (1999). 8-Hydroxydeoxyguanosine and 8-hydroxyguanine as biomarkers of oxidative DNA damage. Methods Enzymol.

[41] Higelin J, Demestre M, Putz S, Delling JP, Jacob C, Lutz AK (2016). FUS Mislocalization and Vulnerability to DNA Damage in ALS Patients Derived hiPSCs and Aging motoneurons. Front Cell Neurosci.

[42] Hill SJ, Mordes DA, Cameron LA, Neuberg DS, Landini S, Eggan K, Livingston DM (2016). Two familial ALS proteins function in prevention/repair of transcription-associated DNA damage. Proc Natl Acad Sci.

[43] Horvath MM, Wang X, Resnick MA, Bell DA (2007) Divergent evolution of human p53 binding sites: cell cycle versus apoptosis. PLoS Genetics 3(7):e127.doi10.1371/journal.pgen.0030127 10.1371/journal.pgen.0030127 PMC1934401 17677004

[44] Imamura K, Izumi Y, Watanabe A, Tsukita K, Woltjen K, Yamamoto T et al (2017) The Src/c-Abl pathway is a potential therapeutic target in amyotrophic lateral sclerosis. Sci Trans Med 9: pii: eaaf3962. 10.1126/scitranslmed.aaf3962 10.1126/scitranslmed.aaf3962 28539470

[45] Kageyama Y, Saito A, Pletnikova O, Rudow GL, Irie Y, An Y (2018). Amyloid β toxic conformer has dynamic localization in the human inferior parietal cortex in absence of amyloid plaques. Sci Rep.

[46] Kamada S, Kikkawa U, Tsujimoto Y, Hunter T (2005). Nuclear translocation of caspase-3 is dependent on its proteolytic activation and recognition of substrate-like protein(s). J Biol Chem.

[47] Kikuchi H, Furuta A, Nishioka K, Suzuki SO, Nakabeppu Y, Iwaki T (2002). Impairment of mitochondrial DNA repair enzymes against accumulation of 8-oxoguanine in the spinal motor neurons of amyotrophic lateral sclerosis. Acta Neuropathol.

[48] Kim SH, Engelhardt JI, Henkel JS, Siklos L, Soos J, Goodman C, Appel SH (2001). Widespread increased expression of the DNA repair enzyme RARP in brain in ALS. Neurology.

[49] Kingma PS, Osheroff N (1997). Apurinic sites are poison-specific topoisomerase II poisons. J Biol Chem.

[50] Kohn KW (1999). Molecular interaction map of the mammalian cell cycle control and DNA repair systems. Mol Biol Cell.

[51] Li Y, Balasubramanian U, Cohen D (2015). A comprehensive library of familial human amyotrophic lateral sclerosis induced pluripotent stem cells. PLoS One.

[52] Lindahl T (1993). Instability and decay of the primary structure of DNA. Nature.

[53] Liu X, Li P, Widlak P, Zou H, Luo X, Garrard WT, Wang X (1998). The 40-kDa subunit of DNA fragmentation factor induces DNA fragmentation and chromatin condensation during apoptosis. Proc Natl Acad Sci.

[54] Liu Z, Martin LJ (2001). Motor neurons rapidly accumulate DNA single-strand breaks after in vitro exposure to nitric oxide and peroxynitrite and in vivo axotomy. J Comp Neurol.

[55] Liu Z, Martin LJ (2001). Isolation of mature spinal motor neurons and single cell analysis using the comet assay of early low-level DNA damage induced in vitro and in vivo. J Histochem Cytochem.

[56] Liu Z, Martin LJ (2003). Olfactory bulb core is a rich source of neural progenitor and stem cells in adult rodent and human. J Comp Neurol.

[57] Lobrich M, Shibara A, Beucher A, Fisher A, Ensminger M, Goodarzi AA (2010). γH2AX foci analysis for monitoring DNA double-strand break repair. Cell Cycle.

[58] Long BH, Musial ST, Brattain MG (1985). Single- and doube-strand DNA breakage and repair in human lung ademocarcinoma cells exposed to etoposide and teniposide. Cancer Res.

[59] Lu T, Pan Y, Kao S-Y, Li C, Kohane I, Chan J, Yankner BA (2004). Gene regulation and DNA damage in the aging human brain. Nature.

[60] Maiani E, Diederich M, Gonfloni S (2011). DNA damage response: the emerging role of c-Abl as a regulatory switch. Biochem Pharmacol.

[61] Mann DMA, Yates PO (1974). Motor neurone disease: the nature of the pathogenic mechanism. J Neurol Neurosurg Psychiatry.

[62] Marsh S (2007). Pyrosequencing applications. Methods Mol Biol.

[63] Martin LJ (1999). Neuronal death in amyotrophic lateral sclerosis is apoptosis: possible contribution of a programmed cell death mechanism. J Neuropathol Exp Neurol.

[64] Martin LJ (2000). p53 is abnormally elevated and active in the CNS of patients with amyotrophic lateral sclerosis. Neurobiol Disease.

[65] Martin LJ (2001). Neuronal cell death in nervous system development, disease, and injury. Int J Mol Med.

[66] Martin LJ (2008). DNA damage and repair: relevance to mechanisms of neurodegeneration. J Neuropathol Exp Neurol.

[67] Martin LJ (2010). Mitochondrial and cell death mechanisms in neurodegeneration. Pharmaceuticals.

[68] Martin LJ, Adams NA, Pan Y, Price A, Wong M (2011). The mitochondrial permeability transition pore regulates nitric oxide-mediated apoptosis of neurons induced by target deprivation. J Neurosci.

[69] Martin LJ, Al-Abdulla NA, Brambrink AM, Kirsch JR, Sieber FE, Portera-Cailliau C (1998). Neurodegeneration in excitotoxicity, global cerebral ischemia, and target deprivation: a perspective on the contributions of apoptosis and necrosis. Brain Res Bull.

[70] Martin LJ, Blackstone CD, Huganir RL, Price DL (1992). Cellular localization of a metabotropic glutamate receptor in rat brain. Neuron.

[71] Martin LJ, Brambrink AM, Price AC, Kaiser A, Agnew DM, Ichord RN, Traystman RJ (2000). Neuronal death in newborn striatum after hypoxia-ischemia is necrosis and evolves with oxidative stress. Neurobiol Dis.

[72] Martin LJ, Chang Q (2018). DNA damage response and repair, DNA methylation, and cell death in human neurons and experimental animal neurons are different. J Neuropath Exp Neurol.

[73] Martin LJ, Chen K, Liu Z (2005). Adult motor neuron apoptosis is mediated by nitric oxide and fas death receptor linked by DNA damage and p53 activation. J Neurosci.

[74] Martin LJ, Kaiser A, Price AC (1999). Motor neuron degeneration after sciatic nerve avulsion in adult rat evolves with oxidative stress and is apoptosis. J Neurobiol.

[75] Martin LJ, Kaiser A, Yu JW, Natale JE, Al-Abdulla NA (2001). Injury-induced apoptosis of neurons in adult brain is mediated by p53-dependent and p53-independent pathways and requires Bax. J Comp Neurol.

[76] Martin LJ, Liu Z (2002). Injury-induced spinal motor neuron apoptosis is preceded by DNA single-strand breaks and is p53- and Bax-dependent. J Neurobiol.

[77] Martin LJ, Liu Z (2002). DNA damage profiling in motor neurons: a single-cell analysis by comet assay. Neurochem Res.

[78] Martin LJ, Liu Z, Chen K, Swaby JA, Golden WC (2007). Motor neuron degeneration in amyotrophic lateral sclerosis mutant superoxide dismutase-1 transgenic mice: mechanisms of mitochondriopathy and cell death. J Comp Neurol.

[79] Martin LJ, Liu Z, Chestnut B, Pipino J, Landek MA (2009). Molecular regulation of DNA damage-induced apoptosis in neurons of cerebral cortex. Cerebral Cortex.

[80] Martin LJ, Pan Y, Price AC, Sterling W, Copeland NG, Jenkins NA (2006). Parkinson’s disease α-synuclein transgenic mice develop neuronal mitochondrial degeneration and cell death. J Neurosci.

[81] Martin LJ, Price AC, McClendon KB, Al-Abdulla NA, Subramaniam JR, Wong PC, Liu Z (2003). Early events in target deprivation/axotomy induced neuronal apoptosis in vivo: oxidative stress, DNA damage, p53 phosphorylation, and subcellular redistribution of death proteins. J Neurochem.

[82] Martin LJ, Wong M (2013). Aberrant regulation of DNA methylation in amyotrophic lateral sclerosis: a new target of disease mechanisms. Neurotherapeutics.

[83] Martin LJ, Wong M (2017). Enforced DNA repair enzymes rescue neurons from apoptosis induced by target deprivation and axotomy in mouse models of neurodegeneration. Mech Ageing & Dev.

[84] Maury Y, Côme J, Piskorowski RA (2015). Combinatorial analysis of developmental cues efficiently converts human pluripotent stem cells into multiple neuronal subtypes. Nat Biotechnol.

[85] Mecocci P, MacGarvey U, Kaufman AE, Koontz D, Shoffner JH, Wallace DC, Beal MF (1993). Oxidative damage to mitochondrial DNA shows marked age-dependent increases in human brain. Ann Neurol.

[86] Miki Y, Swensen J, Shattuck-Eidens D, Futreal PA, Harshman K, Tavtigian S (1994). A strong candidate for the breast and ovarian cancer susceptibility gene BRCA1. Science.

[87] Mitra J, Guerrero EN, Hegde PM, Liachko NF, Wang H, Vasquez V (2019). Motor neuron disease-associated loss of nuclear TDP-43 is linked to DNA double-strand break repair defects. Proc Natl Acad Sci.

[88] Miura M (2012). Apoptotic and nonapoptotic caspase functions in animal development. Cold Spring Harb Perspect Biol.

[89] Morahan JM, Yu B, Trent RJ, Pamphlett (2007) Are metallothionein genes silenced in ALS? Toxicol Lett 168:83-87 10.1016/j.toxlet.2006.11.003 17156946

[90] Morahan JM, Yu B, Trent RJ, Pamphlett R (2009). A genome-wide analysis of brain DNA methylation identifies new candidate genes for sporadic amyotrophic lateral sclerosis. Amyotrophic Lateral Sclerosis.

[91] Moreira MC, Klur S, Watanabe M, Németh AH, Le Ber I, Moniz JC (2004). Senataxin, the ortholog of a yeast RNA helicase, is mutant in ataxia-ocular apraxia 2. Nat Genet.

[92] Moynahan ME, Chiu JW, Koller BH, Jasin M (1999). Brca1 control homology-directed DNA repair. Mol Cell.

[93] Mullaart E, Boerrigter ETI, Ravid R, Swaab DF, Vijg J (1990). Increased levels of DNA breaks in cerebral cortex of Alzheimer’s disease patients. Neurobiol Aging.

[94] Ni X, Yang ZJ, Carter EL, Martin LJ, Koehler RC (2011). Striatal neuroprotection from neonatal hypoxia-ischemia in piglets by antioxidant treatment with EUK-134 or edaravone. Devl Neurosci.

[95] Northington F, Chavez-Valdez R, Martin LJ (2011). Neuronal cell death in neonatal hypoxia-ischemia. Ann Neurol.

[96] Oates N, Pamphlett R (2007). An epigenetic analysis of SOD1 and VEGF in ALS. Amyotrophic Lateral Sclerosis.

[97] Olkowski ZL (1998). Mutant AP endonuclease in patients with amyotrophic lateral sclerosis. NeuroReport.

[98] Prokhorova EA, Kopeina GS, Lavrik IN, Zhivotovsky B (2018). Apoptoosis regulation by subcellular relocation of capases. Sci Rep.

[99] Puthussery T, Fletcher E (2009). Extracellular ATP induces retinal photoreceptor apoptosis through activation of purinoceptors in rodents. J Comp Neurol.

[100] Ranganathan S, Bowser R (2010). p53 and Cell Cycle Proteins Participate in Spinal Motor Neuron Cell Death in ALS. Open Pathol J.

[101] Rao KS (1993). Genomic damage and its repair in young and aging brain. Mol Neurobiol.

[102] Robbins JH (1987). Incorrect priority claim for the DNA damage hypothesis. Arch Neurol.

[103] Rogakou EP, Pilch DR, Or AH, Ivanova VS, Bonner WM (1998). DNA double-strand breaks induce histone H2AX phosphorylation on serine139. J Biol Chem.

[104] Ronen A, Glickman BE (2001). Human DNA repair genes. Environ Mol Mutagen.

[105] Rothstein JD, Martin LJ, Kuncl RW (1992). Decreased glutamate transport by brain and spinal cord in amyotrophic lateral sclerosis. N Engl J Med.

[106] Rowe JB, Toni I, Josephs O, Frackowiak RSJ, Passingham RE (2000). The prefrontal cortex: response selection or maintenance within working memory. Science.

[107] Rowland LP, Shneider NA (2001). Amyotrophic lateral sclerosis. N Eng J Med.

[108] Sau D, De Biasi S, Vitellaro-Zuccarello L, Riso P, Guarnieri S, Porrini M (2007). Mutation of SOD1 in ALS: a gain of a loss of function. Hum Mol Genet.

[109] Schildge S, Bohrer C, Beck K, Schachtrup C (2013) Isolation and culture of mouse cortical astrocytes. J Vis Exp 9:(71). pii: 50079. 10.3791/50079 10.3791/50079 PMC3582677 23380713

[110] Sedelnikov OA, Rogakou EP, Panyutin IG, Boner WM (2002). Quantitative detection of (125)IdU-induced DNA double-strand breaks with γ-H2AX antibody. Radiat Res.

[111] Shaikh AY, Martin LJ (2002). DNA base-excision repair enzyme apurinic/apyrimidinic endonuclease/redox factor-1 is increased and competent in brain and spinal cord of individuals with amyotrophic lateral sclerosis. NeuroMolecular Med.

[112] Shi T, Knaapen AM, Begerow J, Birmili W, Borm PJA, Schins RPF (2003). Temporal variation of hydroxyl radical generation and 8-hydroxy-2’-deoxyguanosine formation by coarse and fine particulate matter. Occup Environ Med.

[113] Stephens B, Guiloff RJ, Navarrete R, Newman P, Nikhar N, Lewis P (2006). Widespread loss of neuronal populations in the spinal ventral horn in sporadic motor neuron disease. A morphometric study. J Neurol Sci.

[114] Sze C-I, Troncoso JC, Kawas C, Mouton P, Price DL, Martin LJ (1997). Loss of the presynaptic vesicle protein synaptophysin in hippocampus correlates with cognitive decline in Alzheimer’s disease. J Neuropathol Exp Neurol.

[115] Taagepera S, McDonald D, Loeb JE, Whitaker LL, McElroy AK, Wang JYJ (1998). Hope TJ. Nuclear-cytoplasmic shuttling of C-ABL tyrosine kinase Proc Natl Acad Sci.

[116] Takahashi G, Sakurai M, Abe K, Itoyama Y, Tabayashi K (2004). MCI-186 reduces oxidative cellular damage and increases DNA repair function in the rabbit spinal cord after transient ischemia. Ann Thorac Surg.

[117] Takizawa Y, Miyazawa T, Nonoyama S, Goto Y-I, Itoh M (2009). Edaravone inhibits DNA peroxidation and neuronal cell death in neonatal hypoxic-ischemic encephalopathy model rat. Pediatr Res.

[118] Tarr IS, McCann EP, Benyamin B, Peters TJ, Twine NA, Zhang KY (2019). Monozygotic twins and triplets discordant for amyotrophic lateral sclerosis display differential methylation and gene expression. Sci Rep.

[119] Tomkins J, Dempster S, Banner SJ, Cookson MR, Shaw PJ (2000). Screening of AP endonuclease as a candidate gene for amyotrophic lateral sclerosis. NeuroReport.

[120] Tomlinson BE, Irving D (1977). The number of limb motor neurons in the human lumbosacral cord throughout life. J Neurol Sci.

[121] Toyokuni S, Iwasa Y, Kondo S, Tanaka T, Ochi H, Hiai H (1999). Intranuclear distribution of 8-hydroxy-2-deoxyguanosine: an immunocytochemical study. J Histochem Cytochem.

[122] Toyokuni S, Sagripanti J-L (1996). Association between 8-hydroxy-2’-deoxyguanosine formation and DNA strand breaks mediated by copper and iron. Free Rad Biol Med.

[123] Toyokuni S, Tanaka T, Hattori Y, Nishiyama Y, Yoshida A, Uchida K (1997). Quantitative immunohistochemical determination of 8-hydroxy-2'-deoxyguanosine by a monoclonal antibody N45.1: its application to ferric nitrilotriacetate-induced renal carcinogenesis model. Lab Invest.

[124] Troncoso JC, Cataldo AM, Nixon RA, Barnett JL, Lee MK, Checler F (1998). Neuropathology of preclinical and clinical late-onset Alzheimer’s disease. Ann Neurol.

[125] Trumbull KA, Beckman JS (2009). A role for copper in the toxicity of zinc-deficient speroxide dismutase to motor neurons in amyotophic lateral sclerosis. Antioxid Redox Signal.

[126] Venkitaraman AR (2001). Functions of BRCA1 and BRCA2 in the biological response to DNA damage. J Cell Sci.

[127] Vodicka P, Stetina R, Polakova V, Tulupova E, Naccarati A, Vodickova L (2007). Association of DNA repair polymorphisms with DNA repair functional outcomes in healthy human subjects. Carcinogenesis.

[128] Wallace SS (1998). Enzymatic processing of radiation-induced free radical damage in DNA. Rad Res.

[129] Wang X, Zeng L, Wang J, Chau JFL, Lai KP, Jia D (2011). A positive role for c-Abl in Atm and Atr activation in DNA damage response. Cell Death Diff.

[130] Wang XD, Zhu MW, Shan D, Wang SY, Yin X, Yang YQ (2019). Spy1, a unique cell cycle regulator, alters viability in ALS motor neurons and cell lines in response to mutant SOD1-induced DNA damage. DNA Repair.

[131] Wang Y, Leung FCC (2004). An evaluation of new criteria for CpG islands in the human genome as gene markers. Bioinformatics.

[132] Watanabe K, Tanaka M, Yuki S, Hirai M, Yamamato Y (2018). How is edaravone effective against acute ischemic stroke and amyotrophic lateral sclerosis. J Clin Biochem Nutr.

[133] Wen Z, Nguyen HN, Guo Z (2014). Synaptic dysregulation in a human iPS cell model of mental disorders. Nature.

[134] Williams AB, Schumacher B (2016). p53 in the DNA-damage-repair process. Cold Spring Harb Perspect Med.

[135] Wong M, Gertz B, Chestnut BA, Martin LJ (2013). Mitochondrial DNMT3A and DNA methylation in skeletal muscle and CNS of transgenic mouse models of ALS. Front Cell Neurosci.

[136] Wong M, Martin LJ (2010). Skeletal muscle-restricted expression of human SOD1 causes motor neuron degeneration in transgenic mice. Hum Mol Genet.

[137] Wood KA, Youle RJ (1995). The role of free radicals and p53 in neuron apoptosis in vivo. J Neurosci.

[138] Wood RD, Mitchell M, Sgouros J, Lindahl T (2001). Human DNA repair genes. Science.

[139] Yang Y, Gozen O, Vidensky S, Robinson MB, Rothstein JD (2010). Epigenetic regulation of neuron-dependent induction of astroglial synaptic protein GLT1. Glia.

[140] Yoshihara M, Jiang L, Akatsuka S, Suyama M, Toyokuni S (2014). Genome-wide profiling of 8-oxoguanine reveals its association with spatial positioning in nucleus. DNA Res.

[141] Zoccolella S, Santamato A, Lamberti P (2009). Current and emerging treatments for amyotrophic lateral sclerosis. Neuropsychiatr Dis Treat.

