# Supplementary figures and images for: DNA damage accumulates and responses are engaged in human ALS brain and spinal motor neurons and DNA repair is activatable in iPSC-derived motor neurons with SOD1 mutations
Source: Acta Neuropathol Commun. 2020 Jan 31;8:7. doi: 10.1186/s40478-019-0874-4 (PMC6995159; doi:10.1186/s40478-019-0874-4)

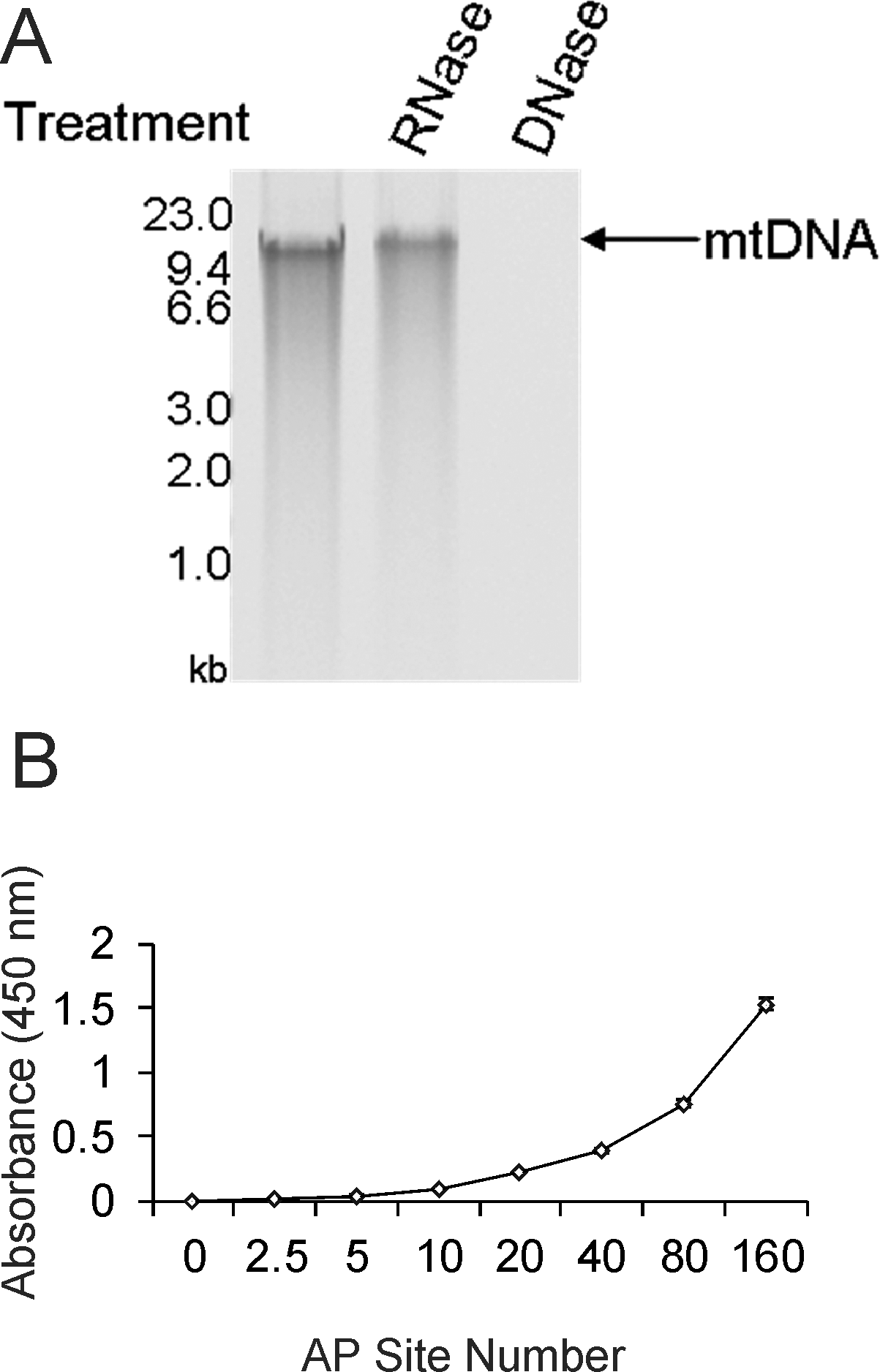

Supplement: Supplementary file 1 — Additional file 1: Figure S1. Validation of mitochondrial DNA purification and DNA-AP site assay sensitivity. A. Agarose gel electrophoresis showing purified DNA isolated from mitochondria of human motor cortex. Band size at approximately 16 kb is consistent with mitochondrial, and the band is RNase insensitive but completely sensitive to DNase. B. Standard curve for the sensitivity of DNA-AP site number. The assay can detect fewer than five AP sites in 1 × 105 bp DNA, and the detection is essentially linear. [file 40478_2019_874_MOESM1_ESM.tif]

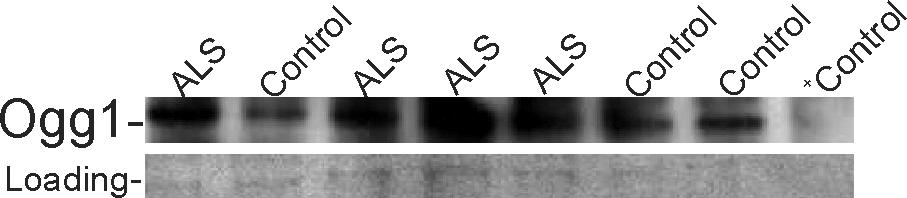

Supplement: Supplementary file 2 — Additional file 2: Figure S2. Western blot for OGG1 proteins levels in human ALS and control motor cortex. Positive control (+control) is human recombinant OGG1. [file 40478_2019_874_MOESM2_ESM.tif]
